# Embryonic Stem Cell Specific “Master” Replication Origins at the Heart of the Loss of Pluripotency

**DOI:** 10.1371/journal.pcbi.1003969

**Published:** 2015-02-06

**Authors:** Hanna Julienne, Benjamin Audit, Alain Arneodo

**Affiliations:** 1 Université de Lyon, Lyon, France; 2 Laboratoire de Physique, CNRS UMR 5672, Ecole Normale Supérieure de Lyon, Lyon, France; The Centre for Research and Technology Hellas, Greece

## Abstract

Epigenetic regulation of the replication program during mammalian cell differentiation remains poorly understood. We performed an integrative analysis of eleven genome-wide epigenetic profiles at 100 kb resolution of Mean Replication Timing (MRT) data in six human cell lines. Compared to the organization in four chromatin states shared by the five somatic cell lines, embryonic stem cell (ESC) line H1 displays (i) a gene-poor but highly dynamic chromatin state (EC4) associated to histone variant H2AZ rather than a HP1-associated heterochromatin state (C4) and (ii) a mid-S accessible chromatin state with bivalent gene marks instead of a polycomb-repressed heterochromatin state. Plastic MRT regions (≲ 20% of the genome) are predominantly localized at the borders of U-shaped timing domains. Whereas somatic-specific U-domain borders are gene-dense GC-rich regions, 31.6% of H1-specific U-domain borders are early EC4 regions enriched in pluripotency transcription factors NANOG and OCT4 despite being GC poor and gene deserts. Silencing of these ESC-specific “master” replication initiation zones during differentiation corresponds to a loss of H2AZ and an enrichment in H3K9me3 mark characteristic of late replicating C4 heterochromatin. These results shed a new light on the epigenetically regulated global chromatin reorganization that underlies the loss of pluripotency and lineage commitment.

## Author Summary

During development, embryonic stem cell (ESC) enter a program of cell differentiation eventually leading to all the necessary differentiated cell types. Understanding the mechanisms responsible for the underlying modifications of the gene expression program is of fundamental importance, as it will likely have strong impact on the development of regenerative medicine. We show that besides some epigenetic regulation, ubiquitous master replication origins at replication timing U-domain borders shared by 6 human cell types are transcriptionally active open chromatin regions specified by a local enrichment in nucleosome free regions encoded in the DNA sequence suggesting that they have been selected during evolution. In contrast, ESC specific master replication origins bear a unique epigenetic signature (enrichment in CTCF, H2AZ, NANOG, OCT4, …) likely contributing to maintain ESC chromatin in a highly dynamic and accessible state that is refractory to polycomb and HP1 heterochromatin spreading. These ESC specific master origins thus appear as key genomic regions where epigenetic control of chromatin organization is at play to maintain pluripotency of stem cell lineages and to guide lineage commitment to somatic cell types.

## Introduction

One of the most remarkable phenomenon in biology is the generation of a whole organism containing a large and phenotypically diverse collection of cells and tissues from a single totipotent cell. This tremendous level of diversity in cellular functions originates from a unique genomic DNA sequence. Since the original sequencing of the human genome a decade ago [1], it has become clear that the functional role of the primary DNA sequence is not only to code for proteins which represent less than 5% of the mammalian genomes, but also to contribute to controlling the spatial structure of DNA in chromatin and in turn to regulate nuclear functions including transcription and replication [2, 3]. But as development goes on, the use of the DNA sequence has to be altered to enable lineage commitment. Epigenetic mechanisms including DNA methylation [4], histone modifications [5–13] and chromatin structure and dynamics [14–25] have been proposed to play a key role in regulation of embryonic development, the maintenance of pluripotency and self-renewal of ESCs, lineage specification and the maintenance of cellular identity during differentiation [26–30]. For years, transcriptional and chromatin changes during mammalian development have been attracting increasing interest. Among noteworthy advances, let us mention the identification of pluripotency markers including NANOG/SOX2/OCT4 [13, 31] and of trithorax proteins and polycomb complexes [32–36] as major actors in developmental gene regulation, the identification of the neural restrictive silencer factor NRSF that represses transcription of several neuronal genes in neural development [37]. Also, as differentiation progresses, chromatin structure switches from a highly dynamic, accessible and permissive euchromatin in ESCs to a less open chromatin riddled with accumulating highly condensed transcriptionally inactive heterochromatin regions [26–28, 38, 39].

In contrast to this overwhelming activity concerning the interplay between chromatin structure and transcription regulation during development, only little attention has been paid to replication and its potential role in lineage commitment and fidelity. There exist however objective reasons to believe that replication may provide some molecular handle on the study of epigenetic programming and reprogramming during development. Specific properties of the ESC cycle such as a high proliferation rate and a shortened G1 phase, that are necessary for self-renewal and the maintenance of pluripotency [29, 40], could explain differences observed between chromatin landscapes and gene expression profiles in pluripotent ESCs and in somatic cells [26–28, 38, 39]. In metazoan genomes, thousand of replication origins are prepared in G1-phase which is more than actively needed in S-phase [41–43]. Epigenetic mechanisms likely take part in the spatial and temporal control of origin usage and efficiency in relation with gene expression [43–47]. In particular, replicon size [48], which is dictated by the spacing between active origins, was shown to correlate with the length of chromatin loops [49, 50] and to be smaller in ESCs than in differentiated cells [51]. DNA replication is also an occasion to act upon the underlying primary chromatin structure at the moment of new histone incorporation or by the spatial reorganization of pre-existent histone marks [45]. The shorter G1-phase and cell cycle duration in ESCs may thus explain the highly dynamic plastic chromatin in pluripotent cells as a lack of time for transcriptionally inactive heterochromatin regions observed in somatic cells to establish [26, 28, 38, 39]. For many years, elucidating the determinants that specify replication program in mammals has been hampered by the limited number of well established origins [43, 46, 52, 53]. The recent availability of genome-wide mean replication timing (MRT) data in various organisms [54, 55], including mouse [56–58] and human [59–62], has given a new impetus to establish links between chromatin structure, transcription and replication [3, 11, 47, 53]. In pioneering studies, in mouse [57, 58] and human [59, 60], replication domains along chromosomes were delineated in constant timing regions (CTRs) of coordinated origin firings and timing transition regions (TTRs) as origin-less regions [63, 64]. In good agreement with previous studies in *Drosophila* [65, 66], these CTRs regions in different mammalian cell types revealed a correlation with epigenetic modifications [67]. Early CTRs tend to be enriched in open chromatin marks, whereas late CTRs are mostly associated with repressive HP1-associated marks [57, 68]. Actually, each cell type presents specific replication timing patterns with mouse ESCs showing a clear MRT pluripotency fingerprint [51]. Differentiation induces important changes in MRT profiles in chromosomal units of size ∼ 400–800 kb [57, 58, 68]. Early to late (EtoL) MRT changes were associated with loss of pluripotency and largely preceded, in development, late to early (L to E) changes associated with germ-layer specific transcriptional activation [68]. Importantly, these dynamic changes in MRT come along with some sub-nuclear repositioning [57, 58, 68–70]. EtoL (resp. LtoE) transitions occur simultaneously with a movement from (resp. towards) interior of the nuclei towards (resp. from) a more peripheral location or near nucleoli [14, 16, 71, 72]. Recent experimental studies of long-range chromatin interactions using chromosome conformation capture techniques [68, 73–75] have confirmed that 3D chromatin tertiary structure plays an important role in regulating replication timing. But as questioned in a detailed analysis of replication fork polarity [76], the above dichotomic picture with early and late replicating loci occurring in separated compartments of open and closed chromatin respectively [68, 73, 74], is somehow a too simple approximation of the information contained in MRT data. The recent analysis of genome-wide MRT data in seven human cell types including ES, somatic and HeLa cells [77, 78], revealed that, in each cell type, about half of the genome can be paved by the so-called replication U-domains where the MRT is U-shaped and its derivative N-shaped like the nucleotide compositional asymmetry in the germline skew N-domains [79–83]. These peculiar N-shaped patterns were shown to be the signature of the existence of large-scale gradients of replication fork polarity [77, 84, 85] originating from transcriptionally active early initiation euchromatin zones (∼200–300 kb) separated by megabase size genome distances [77, 86–89]. These “master” replication origins at U/N-domain borders were further shown to be long-range interconnected hubs of chromatin interactions delineating topological domains of self-interacting chromatin [75, 77, 90]. Here, our aim is to show that, these “master” replication origins at U/N-domain borders [77, 86–91] are a possible clue to the understanding of the plasticity of the spatio-temporal replication program, gene expression and chromatin organization across different cell lines during development, lineage commitment and fidelity.

In a previous study [92], with the aim at quantifying the influence of epigenetic modifications on the spatio-temporal replication program, we used principal component analysis [93] and clustering method [94] to analyze thirteen epigenetic mark maps in the K562 human cell line at the 100-kb resolution of MRT data. This study revealed that the huge combinatorial epigenetic complexity could in fact be reduced to a rather small number of prevalent chromatin states that interestingly shared strong similarities with the ones previously found in *Arabidopsis thaliana* [95], *Caenorhabditis elegans* [96] and *Drosophila* [66, 97]. These four main chromatin states were further shown to replicate at distinct periods of the S-phase, namely from early to late replicating, a gene rich transcriptionally active euchromatin state (C1) enriched in insulator binding protein CTCF, a polycomb repressed facultative heterochromatin state (C2), a silent heterochromatin state (C3) not enriched in any available marks and a gene-poor HP1-associated heterochromatin state (C4). When mapping these chromatin states inside the corresponding megabase-sized U/N-domains, we found that as the signature of an increasing firing frequency during S-phase [98], the accelerating replication wave [76] actually proceeds along a directional path through the four chromatin states, from the open euchromatin state C1 at the “master” replication origins bordering U/N-domains, successively followed by the three silent chromatin states C2, C3 and C4 at U/N-domain centers [92]. The complete analysis of the other half of the human genome that is complementary to U/N-domains [92, 99] turned out to be more consistent with the above mentioned dichotomic picture proposed in pioneering studies of the mouse [56–58] and human [60, 68, 74] genomes. About 25% of the human genome are covered by megabase-sized GC-rich (C1+C2) chromatin blocks that on average replicate early by multiple rather synchronous randomly positioned origins with almost equal proportions of forks coming from both directions which explains that the skew has not accumulated in these gene-rich, high GC isochore-like regions devoid of skew N-domains [79–81]. The last 25% of the human genome corresponds to megabase-sized gene deserts, low GC isochore-like regions of extended (C3+C4) heterochromatin states or long C4 domains that on average replicate late by again multiple almost coordinated origins [92, 99]. Here we extend this study to different cell types including the ESC H1hesc, three hematopoietic cell lines (K562, Gm1278, Monocyte CD14+), a mammary epithelial cell line (Hmec) and an adult fibroblast cell line (Nhdfad). By investigating the global reorganization of replication U/N-domains in these different cell types in relation to coordinated changes in chromatin state and gene expression, we shed a new light on the chromatin-mediated epigenetic regulation of transcription and replication during human differentiation. Because they are likely to be the cornerstone to better understanding of pluripotency maintenance, developmental specification and lineage fidelity, we will pay special attention to the “master” replication initiation zones that border U/N-domains and specially to those that are specific to ESCs.

## Materials and Methods

### Histone marks, H2AZ, CTCF, CHD1, NANOG and OCT4 ChIP-seq data

ChIP-seq data were retrieved for the following cell lines: an ESC line (H1hesc), an immature myeloid cell line (K562), a monocytes-CD14+ (monocd14ro1746), a lymphoblastoid cell line (Gm12878), a mammary epithelial cell line (Hmec), an adult dermal fibroblast cell line (Nhdfad).

For all ChIP-seq data, we downloaded data in the Encode standard format “broadpeaks” (http://genome.ucsc.edu/FAQ/FAQformat.html). Broadpeaks format is a table of significantly enriched genomic intervals. The signal value associated with each enriched intervals is the fold enrichment compared to a uniform distribution of reads [100]. For all cell types, we downloaded the broadpeak tables for the following antibodies: CTCF, H3K27ac, H3K27me3, H3K36me3, H3K4me1, H3K4me2, H3K4me3, H3K9me3, H2AZ, H3K79me2, H4K20me1. For the H1hesc cell line, we downloaded these additional broadpeak genomic intervals: H3K9ac, CHD1, EZH2, NANOG and OCT4.

Most of the data correspond to the release 3 (August 2012) of the Broad histone track, downloaded from: http://hgdownload.cse.ucsc.edu/goldenPath/hg19/encodeDCC/wgEncodeBroadHistone/ The NANOG and OCT4 data corresponds to the release 3 (September 2012) of the HAIB TFBS track. Tables were downloaded from: http://hgdownload.cse.ucsc.edu/goldenPath/hg19/encodeDCC/wgEncodeHaibTfbs/ All these tables in hg19 coordinates were converted to hg18 coordinates using LiftOver.

Note that cell line H1hesc (part of ENCODE Tier 1) is the only embryonic stem cell line for which we could gather a large chromatin mark dataset as described above. Hence, we could not include in the present work the analysis of another ESC line to assess whether the unique properties observed in H1hesc are also valid for other ESC lines.

### Epigenetic profile computation at 100 kb resolution

For each ChIP-seq data and each cell line, we computed a profile at the 100 kb resolution for the 28465 non-overlapping windows corresponding to the sequenced part of the genome. For an antibody, the score in a 100kb window was computed as the sum of the coverage of each significantly enriched interval multiplied by its score; it is thus a read density.

### Construction of a shared epigenetic space for differentiated cell lines

For the five differentiated cell lines (K562, Monocd14ro1746, Gm12878, Hmec and Nhdfad), we constructed a shared epigenetic space. All epigenetic profiles at 100kb of the same mark were concatenated together to obtain one vector of 5 × 28405 = 170970 windows per mark.

### Treatment of H1hesc data set

We took into account the specificity of H1hesc cell line epigenetics by applying the clustering pipeline described in [92] apart from other cell lines but considering the same eleven epigenetic marks as for differentiated cell lines. The number of clusters was set to four because it led to the most qualitatively different chromatin states.

### Rank transformation and Spearman correlation matrix

All statistical computations were performed using the R software (http://www.r-project.org/).

**Figure 1:**
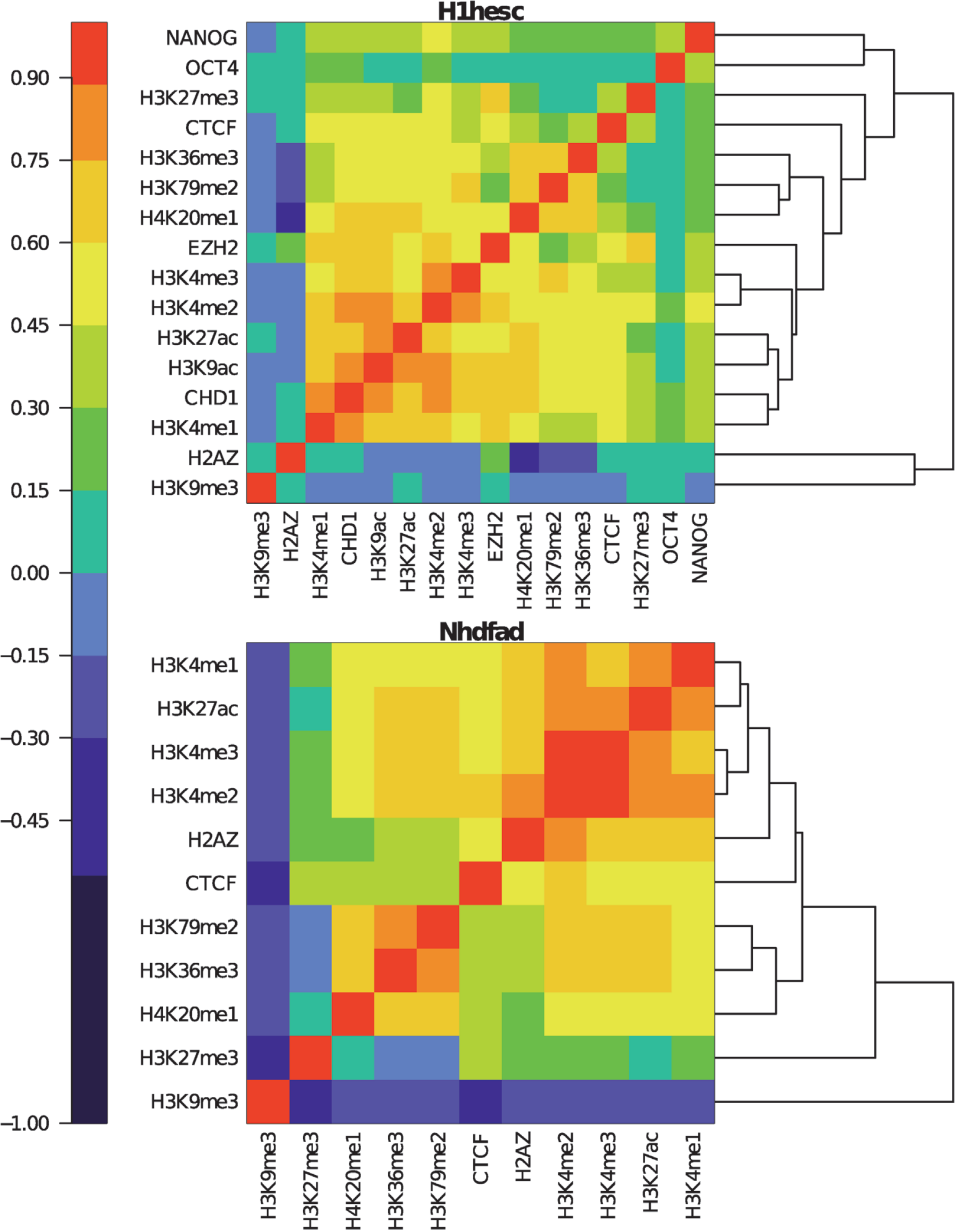
Spearman correlation matrix between epigenetic marks in H1hesc (top) and Nhdfad (bottom). For each cell line, we computed the Spearman correlation over all 100 kb non-overlapping windows with a valid score. Spearman correlation value is color coded using the color map shown on the left. Lines for the epigenetic marks were reorganized by a hierarchical ordering using Spearman correlation distances [Equation (1)] as illustrated by the dendrograms on the right of the corresponding matrices. This ordering implies that highly correlated epigenetic marks are close to each other.

In order to compute the Spearman correlation matrix, the epigenetic profiles at 100 kb resolution were transformed with the R function *rank* with option *ties.method = max*. Then we computed the Pearson correlation matrix on the transformed data set. To reorder the matrix (Fig. 1 and S1 Fig.), we computed the Spearman correlation distance *dSCor* as:

dSCor(X,Y)=1-SCor(X,Y), (1)

where *SCor* is the Spearman correlation. Then, a dendrogram was computed using the R function *hclust* with option *method = average* and with *dSCor* as dissimilarity (Fig. 1). Note that for H1hes cell line, we performed an additional correlation analysis taking into account H3K9ac, CHD1, EZH2, NANOG and OCT4 ChIP-seq data (Fig. 1, top panel).

### Principal component analysis

Principal component analysis was performed on the rank transformed dataset using the function *dudi.pca* from the R package *ade4* (see http://pbil.univ-lyon1.fr/ADE-4 and [101]) with the option *scale = TRUE* (*i.e.* each variable was centered and normalized before the PCA computation). The first four components were retained which accounts for 86% of the dataset variance (S2A and A’ Fig.). Clustering was performed in this 4D space.

### Clustering strategy

We used Clara algorithm [94] which is an optimization of k-means for large data set. We used the *clara* function implemented in the R package *cluster*. The options were set to: *stand = FALSE, sampsize = 500, samples = 20, metric = euclidean*.

For the shared differentiated cells, the number of clusters was set to the number of prevalent chromatin states detected in [92]. Previously to the merging of dataset into one shared epigenetic space, we checked that, when applied to each cell individually, the analysis pipeline led to qualitatively the same epigenetic states (data not shown).

Poorly clustered data points were removed from the set of chromatin states. The silhouette value [102] is a way to quantify how well a point is clustered.

**Definition 1** *Given a particular clustering, C*_1_, *C*_2_, …, *C*_*k*_, *of the data in k clusters, let i be a data point and d*(*i*, *C*_*j*_) *the average distance of the data point i to the members of the cluster C*_*j*_. *Let i be a member of cluster C*_*l*_ *and*

$$a_{i}=d\left(i,C_{l}\right),\;b_{i}=min_{j\neq l}\left(d\left(i,C_{j}\right)\right).$$ (2)

*The silhouette value of the data point i is defined as*:

$$s_{i}=\frac{b_{i}-a_{i}}{max\left(a_{i},b_{i}\right)}.$$ (3)

A silhouette value below 0 means that the data point is actually closer in average to the points from another cluster than to the ones it has been assigned to. Points with a negative silhouette value are questionable assignments. We decided to remove those points from the set of identified chromatin states. Hence chromatin states are groups (clusters) with homogeneous epigenetic features. 91% (resp. 94%) of all 100 kb non-overlapping windows of the human genome were assigned to one of the four chromatin states C1, C2, C3 or C4 (resp. EC1, EC2, EC3 and EC4) in the differentiated (resp. H1hesc) cell lines.

### Mean replication timing data and replication U-domain coordinates

Mean replication timing (MRT) determined in 100 kb non-overlapping windows in hg18 coordinates for an ESC line (BG02), a lymphoblastoid cell line (GM06990), a skin fibroblast cell line (BJ), an immature myeloid cell line K562 and a HeLa cell line were obtained from the authors [77]. The coordinates of the 1534 (BG02), 882 (GM06990), 1150 (BJ), 876 (K562) and 1498 (HeLa) replication U-domains were also obtained from these authors. Replication data in BG02, GM06990 and BJ were used as surrogates of replication data in H1hesc, GM12878 and Nhdfad, respectively. We had previously observed a very strong conservation of MRT between lymphoblastoid cell lines including GM06990 [75], as well as between fibroblast cell lines BJ and IMR90 (unpublished). Note that, due to a lack of data, we do not have evidence of such a conservation between ESC lines.

### DNase Hypersentive site data

DNaseI hypersensitive sites (DHSs) data were downloaded in the Encode standard format “narrowpeaks” (http://genome.ucsc.edu/FAQ/FAQformat.html). DHS narrowpeaks are genomic intervals indentified as hypersentive zones to DNaseI within a false discovery rate of 0.5%. We downloaded the tables from: http://hgdownload.cse.ucsc.edu/goldenPath/hg18/encodeDCC/wgEncodeUwDnaseSeq/

### Annotation and Expression data

As human gene coordinates, we used the UCSC Known Genes table. When several genes presenting the same orientation overlapped, they were merged into one gene whose coordinates correspond to the union of all the overlapping gene coordinates, resulting in 23818 distinct genes.

Expression data were retrieved from the Genome Browser of the University of California Santa Cruz (UCSC). We downloaded expression values from the release 2 of Caltech RNA-seq track (ENCODE project at UCSC): http://hgdownload.cse.ucsc.edu/goldenPath/hg18/encodeDCC/wgEncodeCaltechRnaSeq/

To determine the genome coordinates of each gene (labeled by its RefSeq identifier), we used RefSeq Genes track. For genes associated to more than one splicing variant, we merged exons coordinates by taking their union. Hence the transcription start site (TSS) was placed at the beginning of the first exon. We obtained a table of 23329 genes.

Expression for one gene is given in reads per kilobase of exon model per million mapped reads (RPKM) [103]. RPKM is defined as:

$$R=\frac{10^{9}C}{NL},$$ (4)

where C is the number of mappable reads that fall into gene exons (union of exons for genes with alternative splicing), N is the total number of mappable reads in the experiment, and L is the total length of the exons in base pairs. We associated 17872 genes with a valid RPKM value in K562 and Gm12878 and 17463 in H1hesc.

### CpG o/e computation and GC content

CpG observed/expected ratio (CpG o/e) was computed as $\frac{n_{CpG}}{L-l}\times \frac{L^{2}}{n_{C}n_{G}}$, where *n*_*C*_, *n*_*G*_ and *n*_*CpG*_ are the numbers of C, G and dinucleotides CG, respectively, counted along the sequence, L is the number of nonmasked nucleotides and l is the number of masked nucleotide gaps plus one, *i.e.* L-l is the number of dinucleotide sites. The CpG o/e was computed over the sequence after masking annotated CGIs.

The GC content was computed on the native sequence.

### Nucleosome free regions (NFR)

The coordinates of the NFRs predicted by the physical model defined in [104–107] were obtained from the authors [108]. This theoretical model amounts to compute the energy required for nucleosome formation based on sequence-dependent bending properties [3].

### Chromatin state blocks

We detected contiguous windows of the same chromatin state (C1 to C4 and EC1 to EC4). We then kept the coordinates of the blocks of contiguous windows. To form chromatin state blocks of states (1+2), we simply detected contiguous windows of state 1 or 2. The same procedure was applied to define chromatin blocks of states (3+4). For chromatin blocks (1+2) and (3+4), we authorized the inclusion of isolated windows which did not belong to any chromatin state so to not disrupt very long blocks.

### Replication N-domains

The coordinates of the 678 human replication N-domains for assembly NCBI35/hg17 were obtained from the authors [81] and mapped using LiftOver to hg18 coordinates; we kept only the 663 N-domains that had the same size after conversion [77].

### Index of conservation for U-domain borders

To identify MRT U-domain borders which are common to several cell lines, we constructed a counting signal and we attributed a conservation index as follows:

1. We created a merged data set of the coordinates of all U-domain borders detected in [77] and of skew N-domain borders detected in [81]. U-domains where detected in the following cell line (BG02, K562, GM06990, H0287, TL010, BJ, HeLa). GM06990, H0287, TL010 are three lymphoblastoid cell lines. To avoid lymphoblastoid cell specific U-domains getting an artificially high conservation index, we took only GM06690 into account. To avoid MRT to be a confounding factor, we excluded late U-domain borders with MRT > 0.5.
2. Then, we slided a 200 kb window along the genome with 10 kb incremental steps. At each position, we retrieved the number of cell lines that have a domain border in the window. By doing so, we constructed the counting signal called the conservation index. For instance, if a U-domain border of K562 has a conservation index of 3, it means that this border together with 2 domain borders from other cell lines are contained within a common 200 kb window.

## Results

### Combinatorial analysis of chromatin marks

We investigated relationships between the genome-wide distributions of nine histone modifications, one histone variant and one binding protein at 100 kb resolution in five sommatic cell types including an immature myeloid cell line (K562), a monocyte cell line (Monocd14ro1746), a lymphoblastoid cell line (Gm12878), a mammary epithelial cell line (Hmec), an adult dermal fibroblast cell line (Nhdfad) and an ESC line (H1hesc). As a first step, we computed the Spearman correlation coefficient of each mark with each other (Materials and Methods). We next represented the resulting matrix as a heatmap after having reorganized rows and columns with a hierarchical clustering based on the Spearman distance [Equation (1)] (Fig. 1 and S1 Fig.). This analysis was very enlightening since, on the one hand it revealed that the correlation matrices obtained for the five sommatic cell lines strongly ressemble to the one obtained in K562 in our previous study [92] (Fig. 1, bottom panel and S1 Fig.), and on the other hand it clearly discriminated the pluripotent H1hesc cell line for having a different correlation structure between epigenetic marks (Fig. 1).

In the epigenetic mark matrices obtained for the differentiated cell lines Nhdfad (Fig. 1, bottom panel), Hmec, Monocd14ro1746, K562 and Gm12878 (S1 Fig.), all histone modifications that are known to be involved in transcription positive regulation, namely H3K4me1, H3K4me2, H3K4me3, H3K27ac, H3K36me3, H3k79me2 and H4K20me1, form a block that also includes the histone variant H2AZ and the transcription factor CTCF, meaning that all these marks are all correlated with each other and are likely to occupy similar regions in the genome [6, 12]. In fact, two lines are clearly apart in all correlation matrices as illustrated on the hierarchical clustering dendrogram (Fig. 1, bottom panel). They correspond to the repressive chromatin marks H3K27me3 and H3K9me3 that are respectively associated with the so-called facultative and constitutive heterochromatins [92]. These two marks are recognized by the chromodomains of polycomb (Pc) proteins and heterochromatin protein 1 (HP1) respectively, components of distinct gene silencing mechanisms which may explain that they are anti-correlated with each other. While H3K9me3 behaves quite independently if not anticorrelated with most of the active chromatin marks (except for Gm12878 where some positive correlations were observed), H3K27me3 correlates to some of them in a cell line dependent fashion but more systematically to CTCF and H4K20me1 (Fig. 1, bottom panel and S1 Fig.). This consistency of epigenetic mark correlations in the five differentiated cell lines prompted us to build a “shared” epigenetic space (Materials and Methods). This consisted in pooling data points of all differentiated cell lines together and then in applying PCA and clustering algorithm to reduce the dimensionality of the data. We used the first four principal components along which the data display some meaningful pattern emerging from the noisy background and which together account for 86% of the total data set variance (S2A’ Fig.). As previously experienced with K562 [92], we fixed the number of clusters to four in Clara algorithm [94] (Materials and Methods). When labeling each of the four main chromatin states with a color, we obtained four domains in the (PC1, PC2, PC3, PC4) space that have common boundaries as illustrated on the (PC1, PC2) projection plane (S2B’ and C’ Fig.). To improve the quality of our clustering procedure, we filtered out poorly clustered data points that were closer to another cluster than the one they belong to and had a negative silhouette [102] (Materials and Methods). Note that the classification obtained for K562 in our “shared” epigenetic space is 74% identical to the one previously reported for K562 alone [92]. A similar quantitative identity holds for the four other somatic cell types (data not shown).

The correlation matrix obtained for the same 11 epigenetic mark profiles of the pluripotent H1hesc cell line (Fig. 1, top panel) displays important differences from the ones previously obtained for differentiated cell lines. Among others, let us mention the repressive polycomb-associated mark H3K27me3 which now strongly correlates with most of the active marks including H3K4me3 as the probable signature of bivalent ESC chromatin [6, 9, 26, 28, 32, 33]. Also the histone variant H2AZ that now correlates as much with both the repressive marks H3K27me3 and H3K9me3 as with some of the active marks, which is likely an indication of specific highly dynamic and accessible chromatin of pluripotent cells [6, 9, 11, 26, 28, 39]. When reproducing our PCA and clustering analysis on the H1hesc epigenetic data, we again found that four PCs were enough to account for 86% of the total variance (S2A Fig.), and that one could still reduce the ESC epigenetic complexity to four chromatin states (S2B and C Fig.) but, as described in the next sub-section, these chromatin states are distinct from the ones delineated in sommatic cells confirming that ESCs and differentiated cells have different epigenomes [5, 6, 9, 11, 26, 39]. Note that we have confirmed the conclusions of our correlation matrix analysis (Fig. 1, top panel) when including in our study the distributions of the ATP-dependent helicase CHD1, the EZH2 subunit of polycomb repressive complex 2 (PRC2) and the two pluripotency transcription factors NANOG and OCT4.

### Epigenetic content of prevalent chromatin states in ESCs versus differentiated cells

**Figure 2:**
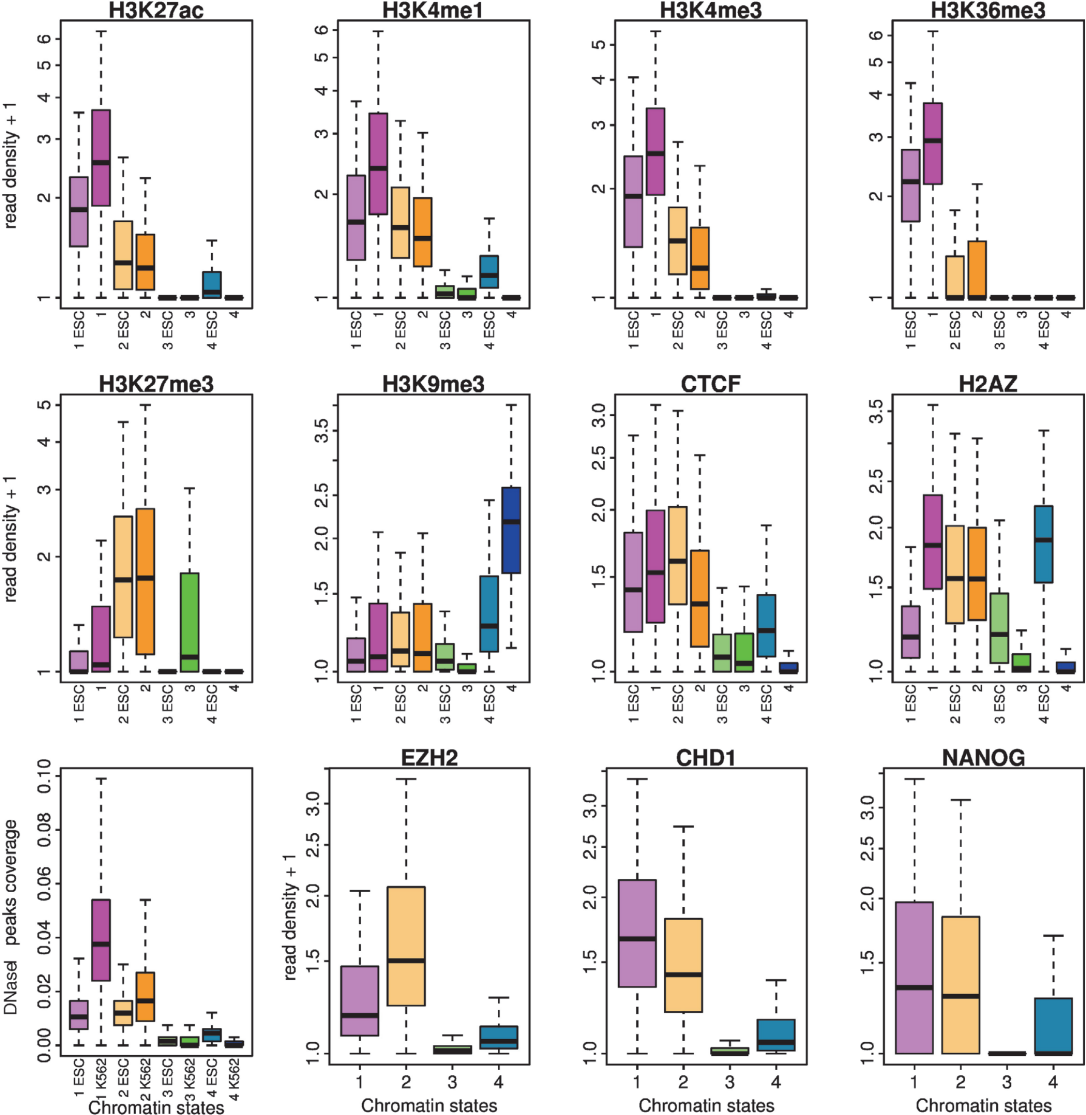
(First two rows) Repartition of epigenetic marks in the four prevalent chromatin states of H1hesc cell line: EC1 (light pink), EC2 (light orange), EC3 (light green), EC4 (light blue) and of differentiated cell lines: C1 (pink), C2 (orange), C3 (green), C4 (blue). Boxplots of the decimal logarithm of histone mark ChiP-seq read density in 100 kb non-overlapping windows per chromatin state. (Third row) Boxplots of the coverage of DNase1 hypersentive peaks in 100 kb non-overlapping windows per chromatin state in H1hesc and K562 cell lines and the decimal logarithm of EZH2, CHD1 and NANOG ChiP-seq read density in 100 kb non-overlapping windows per chromatin state in H1hesc cell lines. Same color coding as above.

The four chromatin states so identified in the five differentiated cell lines are quite similar to the ones previously found in K562 [92] (see also [28]). C1 is a transcriptionally active chromatin state enriched in the histone modifications H3K27ac, H3K4me1, H3K4me3, H3K36me3 (Fig. 2) and H3K4me2, H3K27me2, H4K20me1 (S3 Fig.), as well as in the histone variant H2AZ whose binding level was shown to correlate with gene activity in human [6] (Fig. 2). C2 is notably associated with the histone modification H3K27me3 (Fig. 2) and hence corresponds to a polycomb repressed chromatin state [6, 109]. C3 can be compared to the “null” or “black” silent heterochromatin regions devoid of chromatin marks previously found in *Drosophila* [66, 97] and *Arabidopsis* [95]. C4 corresponds to the HP1-associated heterochromatin state with all C4 100 kb-loci containing H3K9me3 and almost only that repressive mark (Fig. 2) [6, 109]. Note that CTCF which is known to establish chromatin boundaries to prevent the spreading of heterochromatin into transcriptionally active regions [110, 111] was found in C1 and to a slightly less extend in C2 loci (Fig. 2).

Chromatin states in pluripotent H1hesc cell line (EC1, EC2, EC3, EC4) are different even though they display some similarities with the above described differentiated chromatin states (C1, C2, C3, C4). As for differentiated C1 state but to a slightly less extent, more than 75% of 100kb-loci in EC1 state contain all the active histone modification marks considered (Fig. 2, S3 Fig.). 91% of 100 kb loci in EC2 like in C2 are marked by H3K27me3 which is deposited by polycomb complex PRC2 and then enhances PRC1 targeting [35, 36, 112] (Fig. 2). Consistently, EZH2, which is a subunit of PRC2 containing a SET domain that acts upon H3K27 as a methyltransferase, was abundantly found in EC2 confirming the polycomb activity of this state (Fig. 2). CTCF is also present in both EC1 and EC2 as previously seen in C1 and C2 but in slightly reverse importance, EC2 being more enriched than C2 and vice versa for EC1 and C1 (Fig. 2). C1, C2 and EC1, EC2 being the most genic chromatin states in differentiated and ESCs, this result is coherent with the correlation observed between CTCF positioning and gene density [113]. H4K20me1 which was recently shown to strongly correlate with gene activation [6], was consistently found in EC1 and C1 but more surprisingly also in EC2 and C2 which are silent chromatin states (S3 Fig.). Interestingly, PR-Set 7 that is involved in the deposition of H4K20me1, was recently shown to play an important role in the control of replication origin firing in mammalian cells [114].

However the epigenetic chromatin states in pluripotent ESCs and differentiated cells bear more differences than similarities. Systematically the differentiated C1 state is more enriched in active histone marks than the pluripotent EC1 state, and this for all histone modifications but H4K20me1 (Fig. 2 and S3 Fig.). Relatively to EC1, EC2 contains more H3K4me3 than C2 relatively to C1 (Fig. 2), which, with the enrichment of EC2 in H3K27me3, is an indication of bivalent heterochromatin. But the most striking difference concerns the pluripotent state EC4 whose epigenetic content is qualitatively and quantitatively different from the one of C4. Noticeably, H2AZ is highly present in more than 99% of EC4 100kb loci which contrasts with its scarity in C4 (Fig. 2). As compared to C4 which is enriched in the HP1-associated heterochromatin mark H3K9me3, EC4 contains significantly less H3K9me3 concomitantly with an enrichment in H2AZ (Fig. 2). As recently observed in human [28], the enrichment of the ESCs in the histone variant H2AZ associated with nucleosome exchange and remodeling [6, 24, 115, 116] is likely to contribute to the highly dynamic properties of pluripotent chromatin and its refractory character to both HP1- and polycomb heterochromatin extension [6, 11, 26, 28, 38]. This interpretation is stengthened by the observation that in contrast to C4, EC4 is enriched in CTCF (Fig. 2), which besides its insulator properties [110, 111], is also known to mediate long-range intra- and inter- chromosomal interactions [110, 113, 117–120]. Thus, the accessible and more relax EC4 chromatin might be more central in the nucleus than the HP1-associated heterochromatin C4 state that likely corresponds to the emergence of compact chromatin at the nuclear periphery [14–26, 39].

To get a better comprehension of ESC chromatin states, we looked at two additional epigenetic marks known for their implication in pluripotency. Globally all chromatin remodelers are over expressed in ESC [121] but only some knockdown are known to impair pluripotency. The ATP-dependent helicase CHD1 is one of these [122]. In mouse [122, 123], CHD1 helps to maintain a globally more loose chromatin in ESCs. Interestingly, CHD1 is present in EC1 and EC2 (Fig. 2) which makes sense since both these chromatin states contain most of the human genes (discussed below) whose expression can possibly be altered by CHD1 in pluripotent cells [122]. But CHD1 is also present in 84% of EC4 100 kb loci indicating that this remodeler likely contributes to prevent HP1-associated constitutive C4 heterochromatin spreading and compaction [122]. The pluripotent OCT4/SOX2/NANOG network enables self-renewal properties of ESCs, and ectopic expression of these factors together with additional factors or mechanisms was shown to reprogram somatic cells into pluripotent cells (iPS cells) [124, 125]. NANOG was found to the same extend in EC1 and EC2 (Fig. 2) which is consistent with the fact that NANOG regulates roughly the same number of expressed genes and silent genes [31, 126]. NANOG is surprisingly present in the gene-poor EC4 state suggesting that it may play a role in promoting the relative openess of this pluripotent chromatin state.

### Chromatin state coverages and chromatin state changes between cell lines

When comparing the genome coverages, *i.e* the percentages of the 28465 100 kb non-overlapping windows corresponding to the sequenced part of the human genome that belong to the previously identified prevalent chromatin states, we found that whatever the considered cell line, less than 16% of these windows were not properly classified in any chromatin state (Table 1). In H1hesc cells, EC1 and EC2 coverages are about the same (∼ 20%) and are quite similar to the C1 and C2 coverages (∼ 15–23%) generally observed in the five differentiated cells. If the EC3 (27%) and EC4 (24%) coverages are comparable in the ESCs, the C3 and C4 coverages in the differentiated cells are much more variable from 12% to 36%, with a total (C3+C4) coverage ∼ 45% (Table 1).

**Table 1:** Proportions of 28465 sequenced 100-kb windows that belong to the EC1, EC2, EC3 and EC4 chromatin states in H1hesc and to the C1, C2, C3 and C4 chromatin states in differentiated cell lines.

Chromatin states	EC1	EC2	EC3	EC4	ED
H1hesc	0.21	0.19	0.27	0.24	0.09
Chromatin states	C1	C2	C3	C4	D
K562	0.21	0.14	0.28	0.26	0.11
Monocd14ro1746	0.23	0.18	0.13	0.30	0.16
Gm12878	0.21	0.15	0.36	0.12	0.16
Hmec	0.22	0.21	0.25	0.16	0.16
Nhdfad	0.21	0.22	0.19	0.25	0.13

ED and D correspond to 100-kb windows that were not classified in any chromatin states (Materials and Methods).

**Figure 3:**
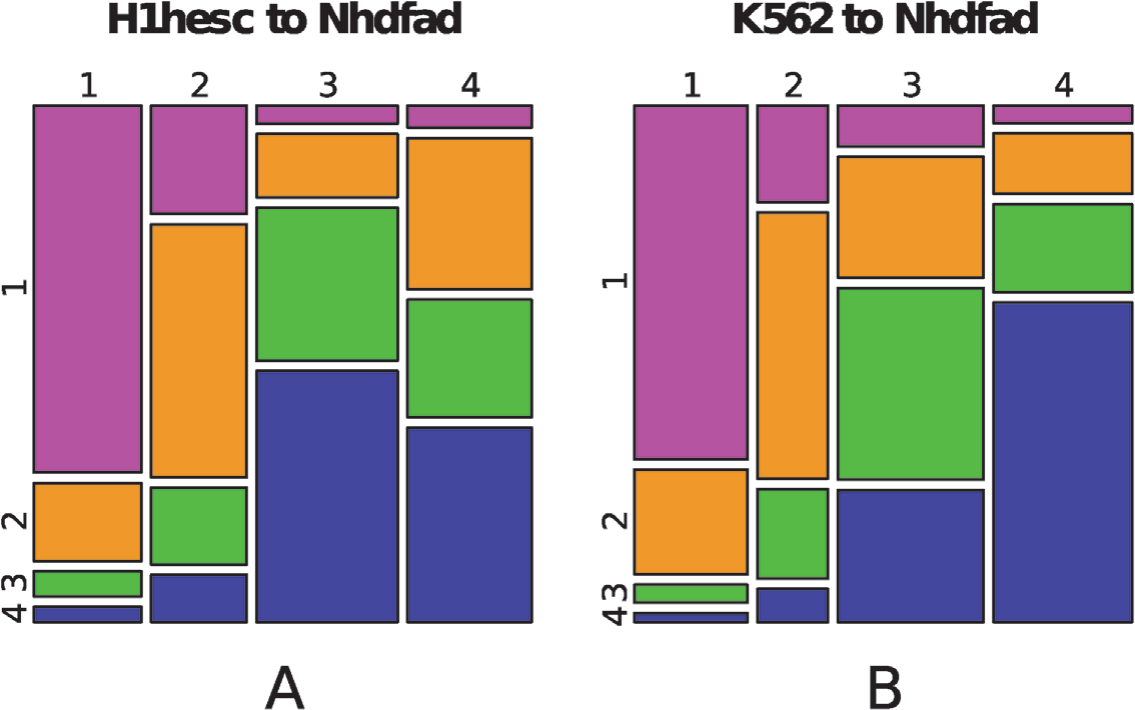
(A) Mosaic plot representing the probabilities of transition from H1hesc chromatin states to Nhdfad chromatin states. The width of columns corresponds to the proportion of chromatin states in H1hesc. The segmentation for the *i* ^*th*^ column follows the proportion of windows in state ECi in H1hec that become Cj in Nhdfad. In other words, if we take the first pink rectangle of the first column, its width is proportional to the probability for a 100 kb window to be in chromatin state EC1 in H1hesc and its height is proportional to the probability for a 100 kb window to be in C1 in Nhdfad given that it is in EC1 in H1hesc. The area of this rectangle (product of the previously mentioned probability) is proportional to the probability for a window to be in state EC1 in ESC and C1 in Nhdfad. (B) Same as (A) for the chromatin state changes from the cell line K562 towards Nhdfad.

To study changes in chromatin states between different cell lines, among all possible pairs of cell lines (S4 Fig.), we focus on two representative transitions from ES to somatic cell lines and from somatic to somatic cell lines. The changes obtained from H1hesc chromatin states to Nhdfad chromatin states (Fig. 3A) reveals that the transcriptionally active state is highly conserved: 80% of EC1 100 kb-loci in H1hesc are C1 loci in Nhdfad as compared to 13% that experience a repression by polycomb to C2 and only 4% and 3% that transit towards the heterochromatin states C3 and C4 respectively. The bivalent state EC2 directs towards either the active euchromatin state C1 (29%) or the polycomb repressed state C2 (51%) which is coherent with initial bivalency adding flexibility in transcription regulation during development [5–12, 33, 127]. The unmarked state EC3 mainly leads to the heterochromatin states C3 (30%) and C4 (51%) and almost never to the active state C1 (5%). EC4 does not transit much to the active state C1 (7%) but distributes almost equally into C2 (34%), C3 (21%) and C4 (39%). Even though they are quite different in terms of epigenetic marks (Fig. 2, S3 Fig.), these three states are silent (Table 2) [92, 99]. Therefore EC4 state in pluripotent cells appears prepared to silencing during differentiation. Now when looking at chromatin state changes from differentiated cell lines K562 to Nhdfad (Fig. 3B), we observed that a majority of 100 kb loci in C1 (73%), C2 (55%), C4 (66%) and to a lesser extend C3 (40%) are conserved. Indeed, a noticeable difference is that the constitutive heterochromatin state C4 rarely transits to the active euchromatin state C1 (4%) and to the polycomb repressed state C2 (12%), which confirms that the pluripotent state EC4, if prepared to silencing, is not as C4, a compactly repressed heterochromatin state. Note that overall, chromatin states are highly dynamic since only 48% (resp. 57%) of 100 kb loci are conserved from H1hesc (resp. K562) to Nhdfad. Merging the genic chromatin states EC1+EC2 (resp. C1+C2) significantly increases the conservation rate to 83% (resp. 69%). The merging of EC3+EC4 (resp. C3+C4) also displays high conservation rate 74% (resp. 89%).

**Table 2:** Gene content in the four prevalent chromatin states of H1hesc and K562 cell lines.

Chromatin states	EC1	EC2	EC3	EC4	C1	C2	C3	C4
promoter count	7653	5047	1138	1505	9588	3089	2334	827
promoter density per Mb	13.127	9.278	1.497	2.16	15.861	7.812	2.953	1.133
mean gene expression (RPKM)	30.537	7.76	0.798	1.299	18.529	2.307	0.696	0.244
median gene expression (RPKM)	8.527	0.988	0.008	0.025	5.58	0.29	0.005	0
mean gene length (kb)	50.999	72.705	56.335	67.538	43.557	58.535	86.301	173.962

For each chromatin state the following information is given: (i) the total number of promoters per chromatin state, (ii) the density of promoters per Mb, (iii) the mean level of expression per chromatin state in RPKM (Materials and Methods), (iv) the median level of expression per chromatin state in RPKM, (v) the mean gene length per chromatin state in kb.

### Replication timing of chromatin states

**Figure 4:**
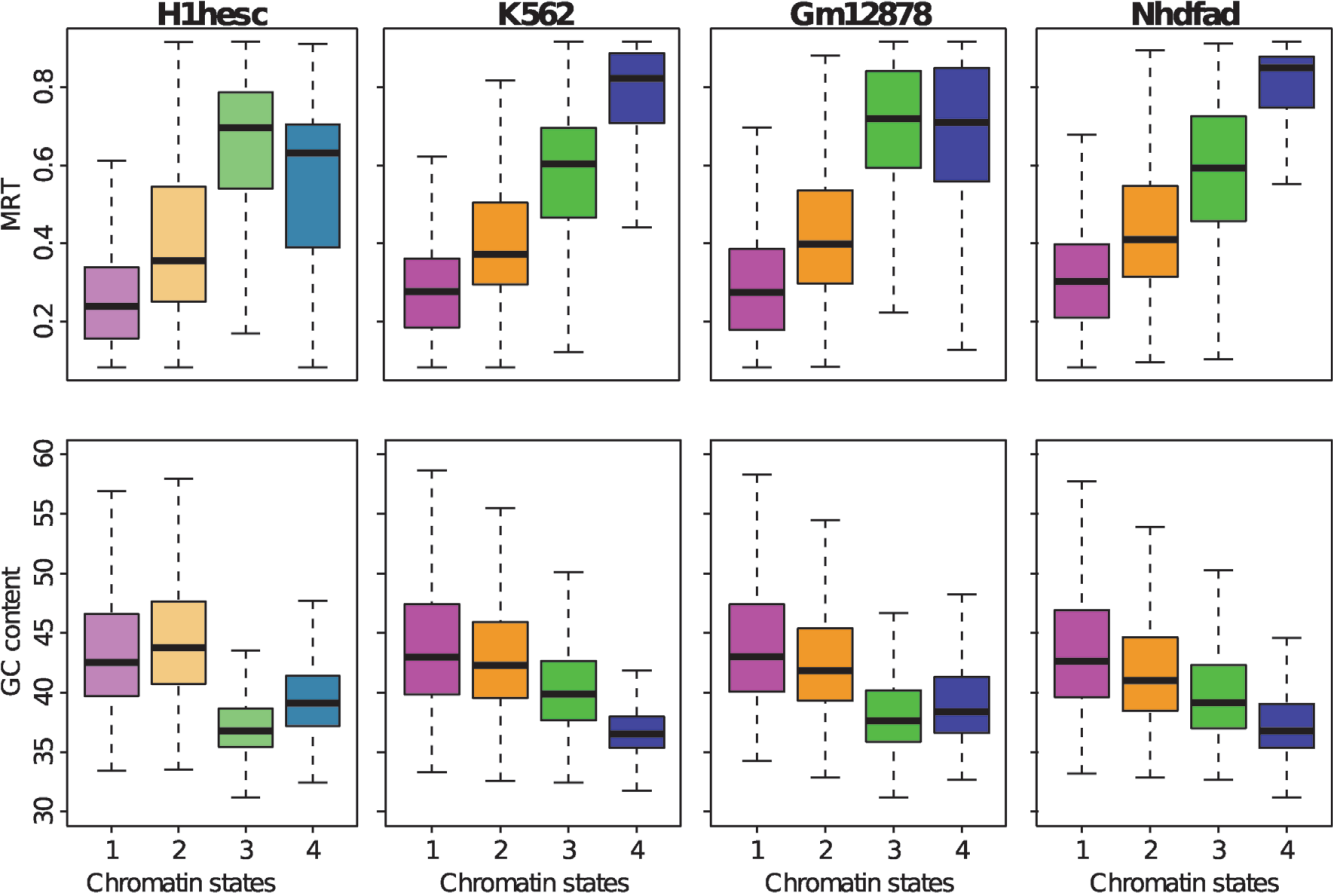
MRT and GC distributions in the four chromatin states for H1hesc and three differentiated cell lines (K562, Gm12878, Nhdfad). (First row) Boxplot of MRT computed in 100 kb non-overlapping windows per chromatin state. (Second row) Boxplots of GC content computed in 100 kb non-overlapping windows per chromatin state. Same color coding as in Fig. 2.

Consistent with our preliminary analysis of the K562 cell line [92], we confirmed that there exists a strong correlation between the four prevalent chromatin states and the MRT, and this for both the pluripotent (H1hesc) and the differentiated (K562, Gm12878, Nhdfad) cell lines (Fig. 4). The transcripionally active euchromatin states C1 and EC1 replicate early in S-phase in agreement with previous studies of open chromatin marks in human and mouse [57, 60, 62, 67, 68, 128]. The pluripotent bivalent EC2 state and the differentiated polycomb repressed C2 heterochromatin state both replicate slightly later in mid-S phase which contrasts with previous report of the existence of high correlation between late replication and the repressive chromatin mark H3K27me3 [68, 129]. The silenced unmarked EC3 and C3 states as well as the pluripotent chromatin state EC4 prepared to heterochromatinization and the HP1-associated heterochromatin state C4 all replicate much latter up to the end of S-phase. Interestingly, whereas (EC1, C1) and (EC2, C2) have clear different MRT, they have almost the same high mean GC content as expected for gene-rich states [1]. In contrast, a clear correlation between MRT and mean GC content was observed for the late replicating chromatin states. When C3 replicates before C4 (K562, Nhdfad), C3 has a higher GC content than C4 and vice-versa when C3 replicates after C4 (H1hesc, Gm12878) (Fig. 4). There is however a major difference between MRT of pluripotent and differentiated cell lines. EC4 exhibits a much wider MRT distribution than C4 with a non-negligible proportion of early replicating (MRT < 0.5) 100-kb loci, namely 35.7% (H1hesc) as compared to 5.5% (K562), 19.2% (Gm12878) and 4.2% (Nhdfad). This can be seen as an additional indication that EC4 is sufficiently accessible and open to enable origin firing and early replication. This is confirmed by non negligible coverage by DNaseI hypersensitive sites (DHS) in H1hesc EC4 (DHS coverage median at 0.45%) as in EC1 (1.1%) and EC2 (1.2%), which contrasts with the abundance of DHS in differentiated C1 (3.8%) and C2 (1.7%) states and their virtual absence in the heterochromatin states C3 (0%) and C4 (0%) (Fig. 2). The highly dynamic and accessible character of pluripotent chromatin states likely facilitates the access of the replication machinery to DNA and thus prevents having to replicate long (EC3+EC4) threads at the end of S-phase.

### Gene content of chromatin states

To address the issue of gene content of pluripotent and differentiated prevalent chromatin states, we focused on H1hesc and K562 cell lines. We took advantage of our previous detailed integrative analysis of epigenetic marks, MRT and gene expression data in K562 [92, 99] that showed that the euchromatin state C1 is highly genic and contains almost all expressed genes and a non negligible proportion of inactive genes that almost equals the total number of genes found in C2 as mostly repressed by polycomb complexes. As compared to these high-GC (Fig. 4), gene rich C1 and C2 states, the low-GC C3 and C4 states were found to be gene deserts with scarce long genes. In the pluripotent H1hesc cell line, the gene rich chromatin states are still EC1 and EC2. But there are some noticeable differences with respect to K562. There are less promoters per Mb in EC1 (13.1 promoters/Mb) than in C1 (15.9 promoters/Mb), and in compensation more in EC2 (9.3 promoters/Mb) than in C2 (7.8 promoters/Mb) (Table 2). Moreover the relative distributions of RPKM values [Equation (4)] (S5A and B Fig.) revealed that relative to C1, EC1 contains more expressed genes with RPKM > 1 as well as EC2 relative to C2. Indeed, both mean and median RPKM values are higher in EC1 and EC2 than in C1 and C2 respectively (Table 2). This is consistent with the extensive presence of bivalent genes in EC2 that was previously shown to be more accessible and less compact than the polycomb repressed C2 state in differentiated cell lines [28, 127]. This is also in agreement with previous report on the higher global transcription activity in ESCs with only sporadic tissue-specific gene expression as compared to differentiated cells [130]. Note that, in that respect, EC4 is slightly permissive to expression whereas C4 is the most repressive heterochromatin state (only 25% of genes with a non-null RPKM) with by far the lowest gene density and largest gene mean length (Table 2).

The coupling between MRT and gene expression has been extensively studied in *Drosophila* [55, 65] and mammals [56, 57, 59, 60]. We found that in both H1hesc and K562, a vast majority of expressed genes are in the early replicating EC1 and C1 chromatin states which confirms the link between MRT and expressed gene density previously reported in mouse [56–58] and human [59, 60, 62, 92]. Even more, in [99] we showed that the activation of one gene in K562 was almost always sufficient for its 100 kb environment to be in a early C1 chromatin state. But the presence of an important number of inactive genes in early C1 regions and to a less extend in early EC1 regions (Table 2, S5A and B Fig.), suggests that there is no causal link between an early replicating region and a high expression level yet many recently identified early replication origins are strongly associated with CGI and active CpG-rich gene promoters [43, 47, 131–139]. If almost all genes in the late replicating heterochromatic C3 and C4 states are silent with few exceptions, there is a slighlty larger number of expressed genes in the pluripotent EC4 state (25% of the few genes in EC4 100kb windows have a non null RPKM). Recent studies in mammals have further shown that the dynamic of MRT through differentiation is only loosely coupled with gene expression dynamic [57, 58, 60, 65].

### Spatial organization of chromatin states along human chromosomes

**Figure 5:**
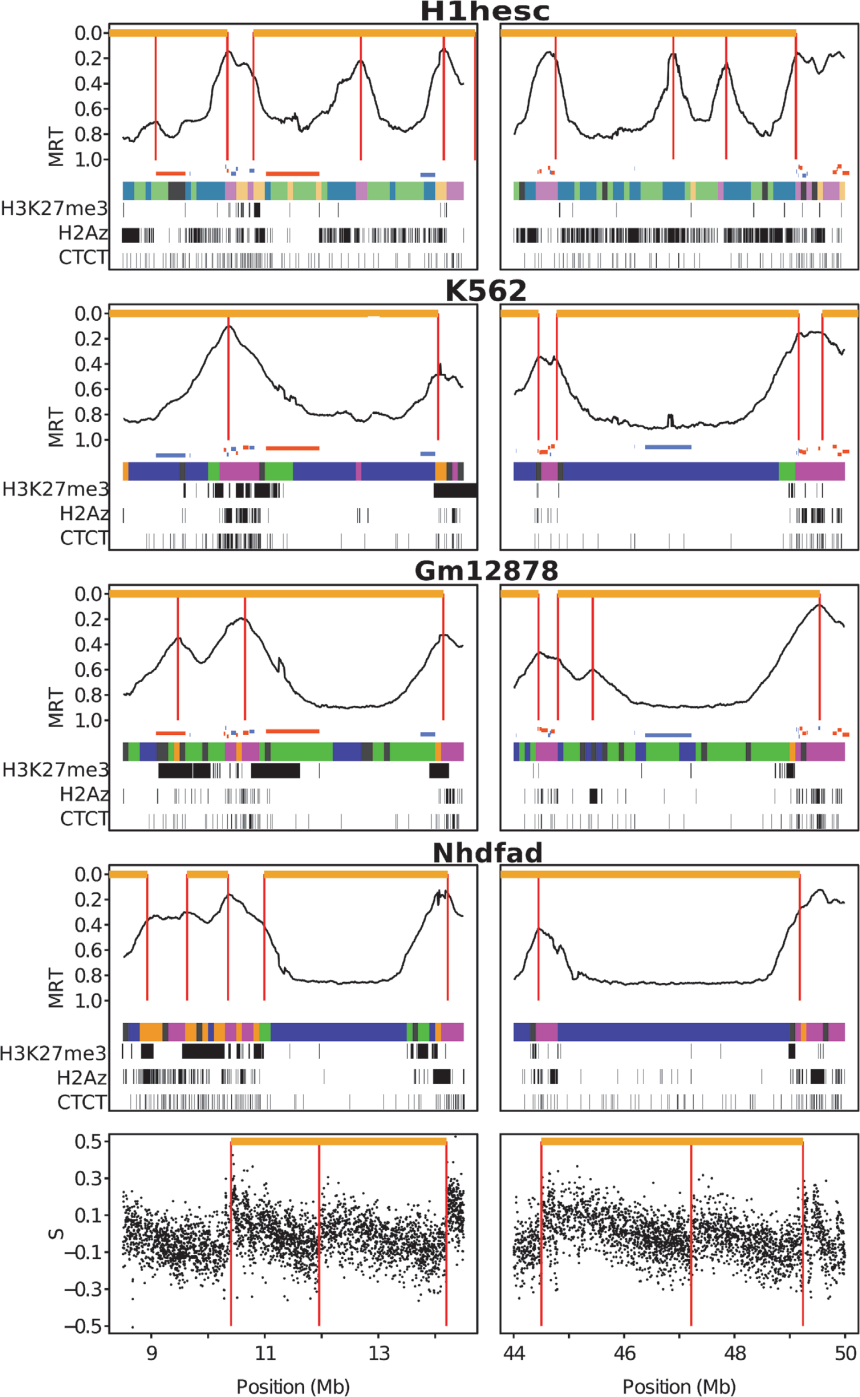
Genome-wide spatial distribution of chromatin states in ESCs and differentiated cells. MRT profile along two Mb long fragments of human chromosome 5 (left) and 14 (right). U-domains are marked by an horizontal orange line and their borders by vertical red lines. Below the MRT profile, gene positions are indicated by a horizontal segment (blue: not expressed, orange: expressed) as well as the chromatin state of each 100 kb window is represented using the same color coding as in Fig. 2. At the bottom of the plot, intervals significantly enriched in H3K27me3, H2AZ and CTCF are represented in black. At the bottom of the figure, the last panel represents the compositional skew $S=S_{GC}+S_{TA}=\frac{G-C}{G+C}+\frac{T-A}{T+A}$ [79–82] in 1 kb windows of repeat masked sequences; germline replication skew N-domains are marked by an horizontal orange line and their borders by a vertical red line.

Once mapped on the genome (Fig. 5), the organization of the four prevalent chromatin states looks different in the pluripotent H1hesc cell line as compared to the one in the five differentiated cell lines (Table 3). In H1hesc, the four chromatin states EC1, EC2, EC3 and EC4 do not differ so much in their genome coverage (Table 1). Moreover, the blocks of adjacent 100-kb-loci in the same chromatin state have similar length distributions (Table 3, S6A Fig.). In Nhdfad, in agreement with previous analysis in K562 [92], the HP1-associated heterochromatin state C4 has a block length distribution that displays a fat tail not observed in the C1, C2 and C3 block length distributions (S7A Fig.) nor in the corresponding H1hesc block length distributions (S6A Fig.). This fat tail explains that the mean C4 block lengths ($\bar{\text{L}}=894$ kb) is significantly larger than the mean block length of C1 ($\bar{\text{L}}=312$ kb), C2 ($\bar{\text{L}}=262$ kb) and C3 ($\bar{\text{L}}=281$ kb) (Table 3). This peculiar length property of C4 blocks is shared by all differentiated cell lines except Gm12878 where C3 blocks are larger ($\bar{\text{L}}=576$ kb) as compared to C4 blocks ($\bar{\text{L}}=276$ kb). Interestingly, as originally observed in K562 [92], for all differentiated cell lines as well as for the ESC line H1hesc, the association of C1+C2 (resp. EC1+EC2) on one side and C3+C4 (resp. EC3+EC4) on the other side, results in large scale blocks of surprisingly similar length distributions (Table 3, S6B and S7B Figs.). The block length distributions obtained for differentiated cells have a fat tail up to blocks larger than 10 Mb (S7B Fig. and also Fig. 9 in [92]). These very long C1+C2 blocks actually replicate very early (S7C Fig.). Within these long blocks, C2 loci replicate later than C1 loci suggesting that C2 loci are replicated passively from fork coming from neighboring C1 active loci. On the contrary, long C3+C4 blocks replicate very late (S7D Fig.) as expected for gene desert low-GC heterochromatin regions. These results are quite consistent with the statistical model proposed in [60] where MRT is predicted from the distance to the nearest active promoter. In H1hesc, the long EC1+EC2 (resp. EC3+EC4) blocks also correspond to early (reps. late) replicating regions (S6C and D Figs.). Interestingly, their maximal length (∼ 5Mb) is significantly shorter than in differentiated cells (∼12 Mb), which might be related to a shorter cell cycle in ESC.

**Table 3:** Mean length of chromatin state blocks per chromatin state in kb (Materials and Methods) in ES H1hesc and differentiated cells (see Table 1).

Chromatin state	EC1	EC2	EC3	EC4	EC1+EC2	EC3+EC4
H1hesc	296.14	241.89	398.56	300.81	569.34	740.2
Chromatin state	C1	C2	C3	C4	C1+C2	C3+C4
K562	327.43	191.14	437.69	881.56	567.19	869.6
Monocd14ro1746	357.48	215.22	210.33	582.15	642.93	628.52
Gm12878	336.87	198.34	576.08	276.3	551.83	702.88
Hmec	322.34	231.96	357.57	610.08	608.46	563.51
Nhdfad	312.11	261.89	281.14	894.38	675.45	637.58

### Mean replication timing dynamics during differentiation

**Figure 6:**
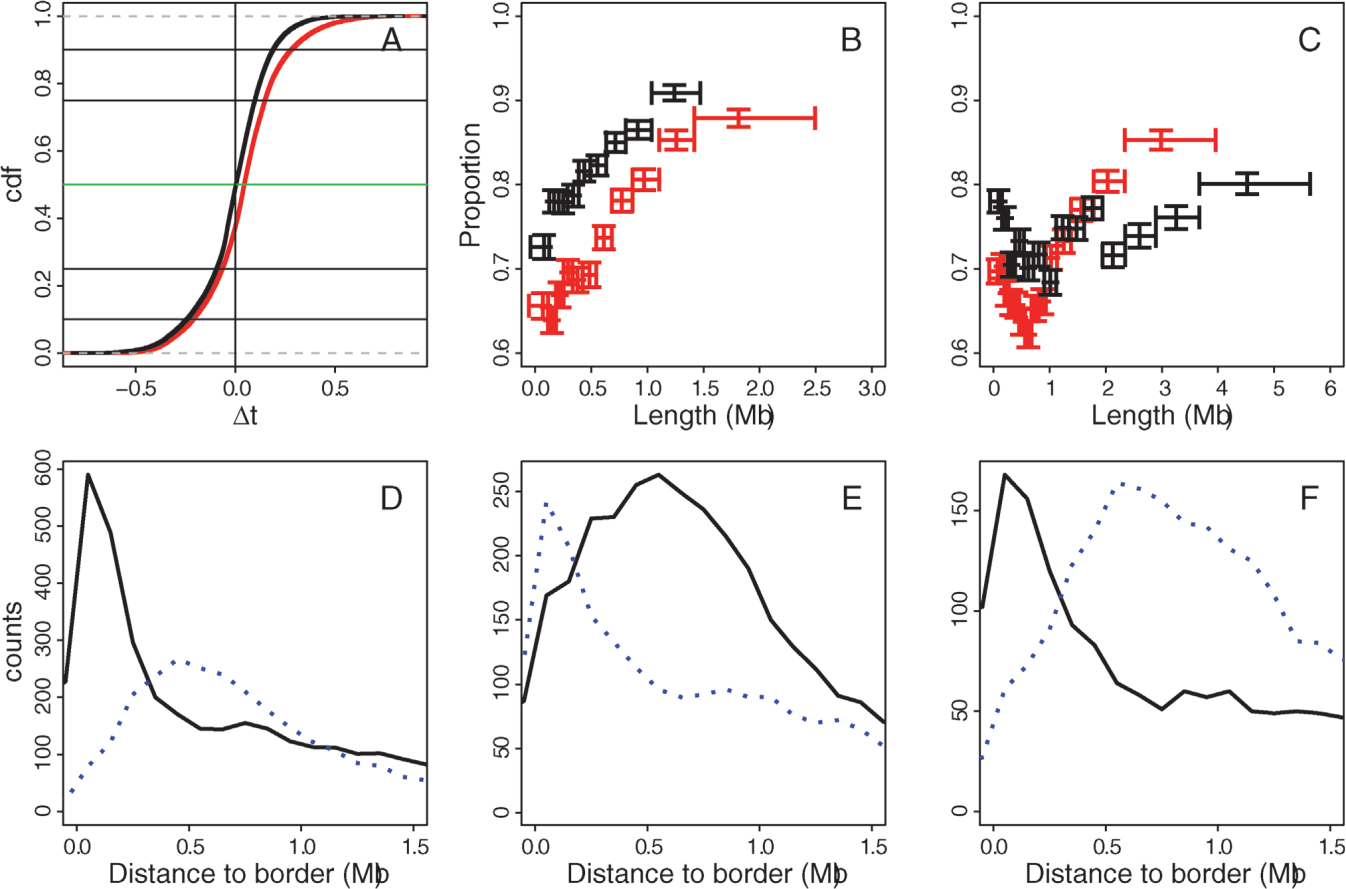
(A) Cumulative distribution function (cdf) of MRT difference Δ*t* between two cell lines: Δ*t* = MRT(Nhdfad) − MRT(H1hesc) (red) and Δ*t* = MRT(Nhdfad) − MRT(K562) (black). Horizontal lines mark from bottom to top the first decile, first quartile, median, last quartile and last decile, respectively. (B) Proportion of 100 kb windows in C1+C2 blocks that have a conserved MRT (∣Δ*t*∣ < 0.2) with respect to block length: H1hesc blocks with MRT compared to Nhdfad (red) and K562 blocks with MRT compared to Nhdfad timing (black). (C) Same as (B) for C3+C4 blocks. (D) Histogram of distances to H1hesc U-domain borders for loci that have a Δ*t* = MRT(Nhdfad) − MRT(H1hesc) > 0.2 (line) and < −0.2 (dots). (E) Histogram of distances to Nhdfad U-domain borders for loci that have a Δ*t* = MRT(Nhdfad) − MRT(H1hesc) > 0.2 (line) and < −0.2 (dots). (F) Histogram of distances to K562 U-domain borders for loci that have a Δ*t* = MRT(Nhdfad) − MRT(K562) > 0.2 (line) and < −0.2 (dots).

Consistently with previous studies in mouse [57, 58], most 100 kb loci (≳ 80%) do not present significant MRT change (|Δ*MRT*| < 0.2) when comparing two cell types (Fig. 6A). Whereas there are as many positive (EtoL) as negative (LtoE) MRT changes between two somatic cell types, there is an excess of EtoL transitions from H1hesc to somatic cell types (Fig. 6A). The MRT conservation level of C1+C2 (EC1+EC2) chromatin blocks (Fig. 6B) and of C3+C4 (EC3+EC4) chromatin blocks (Fig. 6C) is clearly larger for the largest blocks. For the former, the conservation level increases with the size of the chromatin block indicating that mostly small C1+C2 (EC1+EC2) fragments a few hundred kb long switch MRT from one cell type to another. In contrast, MRT conservation level for small C3+C4 (EC3+EC4) presents a minimum around ∼ 600 kb. This means that not only the largest but also the very small blocks have a robust MRT between cell types. These small C3+C4 (EC3+EC4) blocks have a similar conservation level as their surrounding C1+C2 (EC1+EC2) blocks that likely contribute to stabilize their MRT via passive replication [140, 141].

Altogether, the largest chromatin state blocks correspond to mega-base sized MRT domains that are well conserved between pluripotent and differentiated cell lines (Fig. 6B and C). These constant MRT regions cover about half of the human genome in agreement with the dichotomic view proposed in early studies of the mouse [56–58] and human [60, 68, 74] genomes, where early and late replicating regions occur in separated compartments of open and close chromatin, respectively. About 25% of the human genome are covered by megabase sized gene-rich, high-GC EC1+EC2 (resp. C1+C2) chromatin blocks in H1hesc (resp. differentiated) cells, that on average replicate early (S6C and S7C Figs.). Since the replication fork polarity is reflected in the MRT derivative [77, 84], each locus along these MRT plateaus is replicated by an equal proportion of forks coming from both directions originating from multiple early firing origins. Similarly, about 25% of the genome are covered by megabase sized gene-poor, low-GC EC3+EC4 (resp. C3+C4) chromatin blocks in H1hesc (resp. differentiated) cells, that on average replicate late (S6D and S7D Figs.) by again multiple almost coordinated origins. Note that despite the difference in chromatin properties of the silent states EC4 (dynamically accessible) and C4 (compact heterochromatin), these late MRT plateaus present a similar MRT conservation level as the early EC1+EC2 (resp. C1+C2) plateaus.

In all the cell lines examined in this work, the other half of the human genome complementary to the mega-base sized early and late MRT domains was shown to be paved by U-shaped MRT domains (Fig. 5) [77]. However their number (N) and mean length ($\bar{\text{L}}$) drastically differ in H1hesc (N = 1534, $\bar{\text{L}}=1.09$ Mb) and in the differentiated cell lines K562 (N = 876, $\bar{\text{L}}=1.42$ Mb), Gm12878 (N = 882, $\bar{\text{L}}=1.52$ Mb) and Nhdfad (N = 1150, $\bar{\text{L}}=1.19$ Mb). MRT U-domains are more numerous and shorter in the ESC line than in the differentiated cell lines. The corresponding excess of early replicating U-domain borders in H1hesc possibly underlies the excess of EtoL transitions observed from H1hesc to somatic cell types (Fig. 6A). Interestingly, if these MRT U-domains are a robust feature of the spatio-temporal replication program in human, they indeed correspond to the most dynamical regions of the genome during differentiation for MRT changes [77]. The 100 kb loci that present an EtoL MRT switch from H1hesc to Nhdfad are preferentially located (resp. depleted) within ±300 kb of replication U-domain borders in H1hesc (resp. Nhdfad) (Fig. 6D and E). MRT plasticity is thus concomitant with the disappearance of MRT U-domain borders consistently with the replication consolidation scenario previously reported in mouse [57, 58]. An active early replication initiation zone in ESCs that no longer fires early results in the merging of two neighboring MRT U-domains in H1hesc into a larger MRT U-domain in Nhdfad. In contrast, the situation is the opposite for the 100 kb loci that present an LtoE MRT switch from H1hesc to Nhdfad that actually corresponds to the breaking of one H1hesc U-domain into two Nhdfad U-domains via the appearance of an early initiation region (Fig. 6D and E). Note that a similar U-domain border dynamic is also observed between somatic cell types (Fig. 6F).

### Chromatin state organization inside replication U-domains

Replication U-domains were detected as regions bordered by two early replicating regions having a U-shaped MRT profile [77, 78], so that these domains capture the spatial coherence of MRT distribution along the genome. In this respect, we reasoned that mapping the organization of the four prevalent chromatin states within replication U-domains can provide complementary (but not independent) information on the genomic organization of chromatin states and on the modifications of this organization during cell differentiation, compared to the results described above about the spatial distribution and the replication timing of chromatin states.

**Figure 7:**
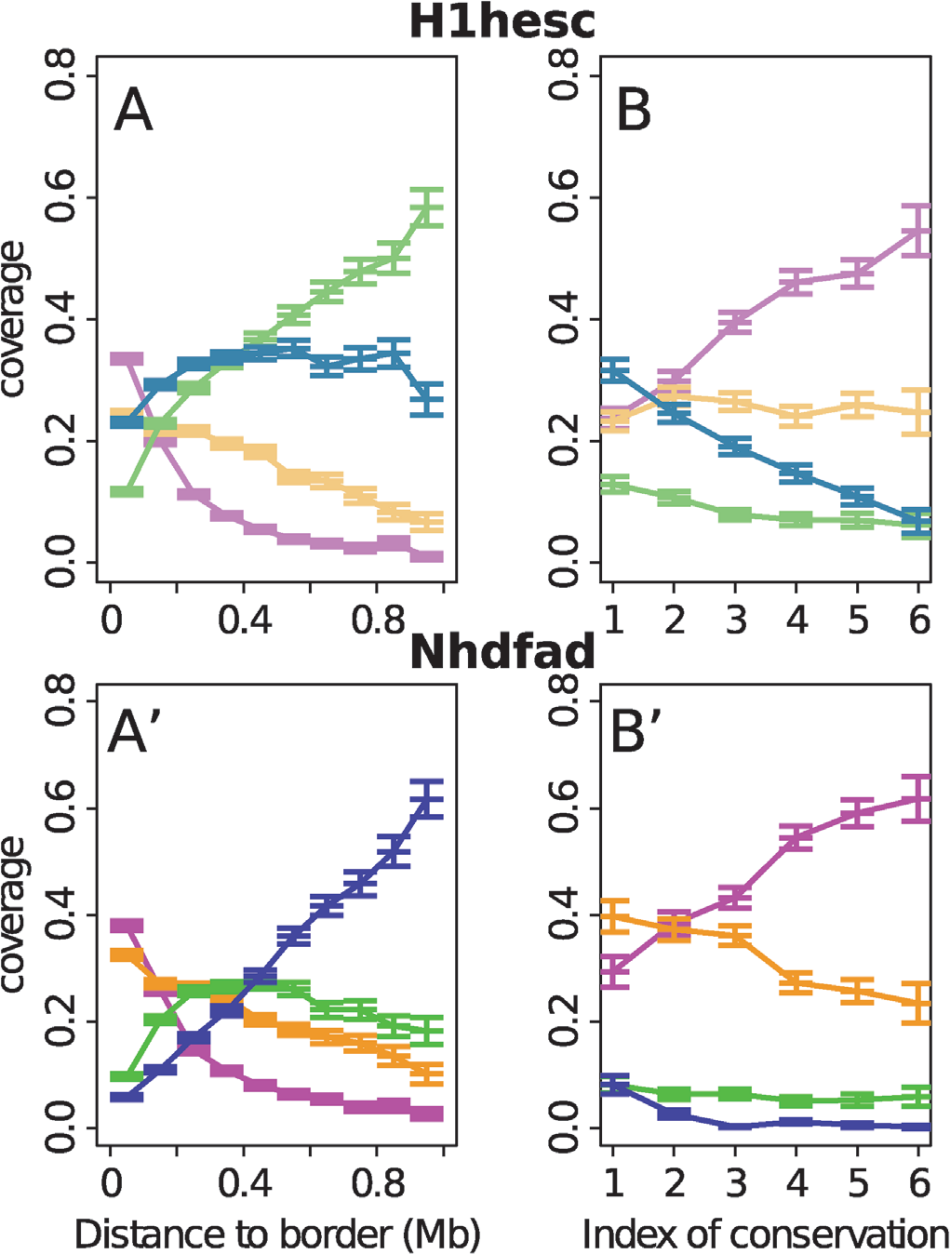
Distribution of chromatin states inside replication timing U-domains of H1hesc and Nhdfad. (A) Mean coverage of chromatin states with respect to the distance to the closest U-domain border in the H1hesc cell line. (B) Mean coverage of ESC chromatin state in the 100kb window containing a U-domain border with respect to the conservation index n of the U-domain border (Materials and Methods). (A’, B’) same as (A, B) for the Nhdfad cell line. Same color coding as in Fig. 2.

**Figure 8:**
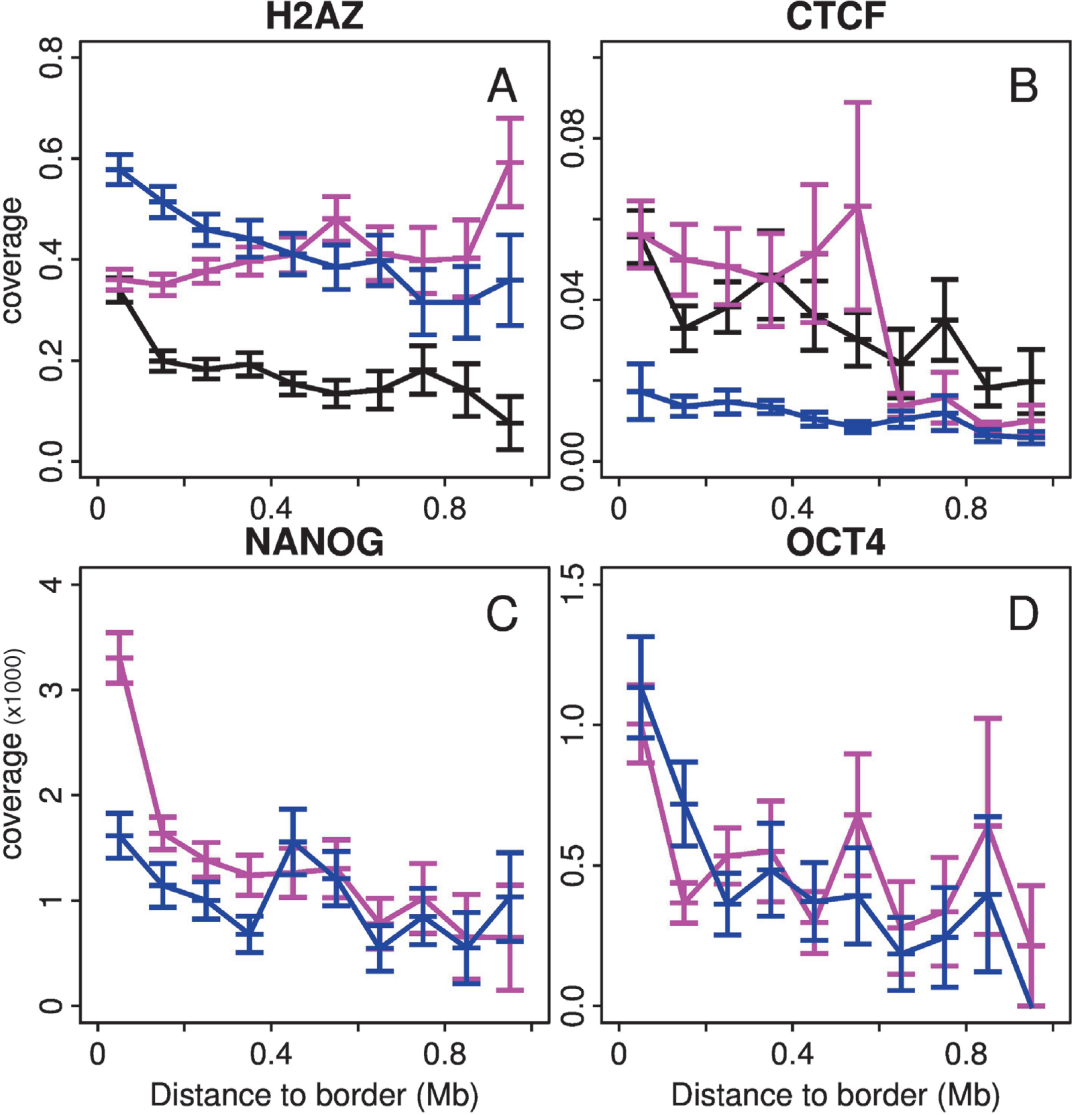
Epigenetic marks enrichment in specific MRT U-domains of H1hesc and Nhdfad. (A) Mean coverage of H2AZ enriched intervals with respect to the distance to the closest U-domain border specific to the cell line. The different colors correspond to specific U-domains of Nhdfad (black), specific U-domains of H1hesc whose border is in EC1 or EC2 (red) and specific U-domains of H1hesc whose border is in EC4 (blue). (B), (C) and (D) are as (A) for respectively CTCF, NANOG anf OCT4. Note that in (C) and (D) coverage are per thousand.

When concentrating our study on the replication U-domains identified in H1hesc (Fig. 7A and B) and Nhdfad as a representative of differentiated cell lines (Fig. 7A’ and B’), we revealed some remarkable organization of the four prevalent chromatin states with some notable differences that distinguish the global dynamical and accessible character of pluripotent chromatin from the expanding HP1-associated heterochromatin in differentiated cells. Consistent with the organization found in K562 [92], the highly expressed gene-rich open euchromatin state C1 was found to be confined in a closed (≲ 150 kb) neighborhood of the master replication origins that border each individual U-domains (Fig. 7A’) and this independently of the domain size [92] (data not shown). Significantly enriched in DHS and CTCF (Figs. 2 and 8B), C1 can thus be seen as specifying the early initiation zones that border U-domains and that were further shown to delimit topological domains on genome-wide (4C, Hi-C) chromatin state conformation data [75, 77, 90]. The polycomb repressed state C2 was mainly found occupying the mid-S phase 200–300 kb region away from U-domain borders (see also Fig. 9 in [92]). Remarkably, U-domain borders are significantly depleted in unmarked (C3) and constitutive (C4) heterochromatin states (Fig. 7A’). C3 homogeneously occupies large U-domain centers. C4 is abundantly found in the center of large U-domains (≳ 1Mb). These results for Nhdfad and K562 [92] suggest that a replication “wave” starting from the early initiation zones at U-domain borders and propagating inside these domains via the progressive activation of secondary origins [76, 89], actually progress in a gradient of chromatin structures from openess (C1) to compactness (C3, C4), via the polycomb repressed state C2 [92].

In the smaller H1hesc U-domains, the concentration of EC1 around the bordering master replication initiation zones and the distribution of EC2 nearby in mid-S phase proximal regions (Fig. 7A) resembles to the organization of high-GC, gene-rich chromatin states (C1, C2) in differentiated cells (Fig. 7A’). However the distributions of EC3 and EC4 (Fig. 7A) are different from those of C3 and C4 in Nhdfad (Fig. 7A’) and K562 [92]. EC3 is still depleted at U-domain borders and mainly covers the center of the largest U-domains. Importantly, unlike C4, EC4 is now found at many U-domain borders as well as inside these domains. As addressed in the “Discussion”, this homogeneous distribution of the gene-poor silent EC4 state inside replication U-domains actually reflects the almost uniform covering (inside U-domains as well as outside) of the human genome by the histone variant H2AZ in pluripotent cells (Fig. 8A) [28].

## Discussion

### Specific genome-wide histone signature of pluripotent plastic chromatin

Our integrative analysis of epigenetic marks confirmed the existence of fundamental differences between the pluripotent and differentiated chromatin states (Fig. 2). These differences account for the changes observed in epigenetic landscapes in ESC and lineage committed cells (Fig. 5) [6, 9, 11, 26, 28, 39]. In general, histone modifications show two distinct types of spatial distributions: small localized peaks and large spreading domains. The histone variant H2AZ associated with nucleosome exchange and remodeling [6, 11, 23, 24, 28, 115, 116], was typically found confined to promoters and distal elements in differentiated cells [6, 28] which explains its abundance in the gene-rich chromatin states C1 and C2 (Fig. 2). Its binding level was further shown to correlate with gene expression in human [6] which is consistent with its highest enrichment in the transcriptionally active state C1. Remarkably, the global H2AZ distribution diverges markedly between pluripotent and differentiation cells. In H1hesc, 92% of the overall 100-kb loci contain the H2AZ mark as compared to smaller coverages in K562 (61%), Gm12878 (65%), Hmec (76%) and Nhdfad (79%) (Note the important covering found for Monocd14ro (94%)) (S1 Table). Thus in ESCs, H2AZ marks promoters and distal elements but it is also distributed thoughout intergenic regions which explains its presence in the gene-rich chromatin states EC1 and EC2 and in addition, its specific abundance in the gene-poor chromatin state EC4 (Fig. 2). This broad H2AZ distribution suggests that chromatin exchange and remodeling are prevalent throughout human chromosomes in ESCs [28]. This highly dynamic and potentially accessible properties of pluripotent chromatin are further strengthened by the presence of the ATP-dependent remodeler CHD1 not only in EC1 and EC2 but also in EC4 as an inhibitory factor to HP1-heterochromatin (Fig. 2) [122]. In addition, we showed that the active marks H3K4me1 has a broad dispersion in H1hesc (85% coverage) as compared to differentiated cells types K562 (55%), Monocd14ro1746 (61%), Gm12878 (62%), Hmec (77%) and Nhdfad (72%) (S1 Table), likely resulting from a more restrictive confinement at promoters and enhancers in differentiated cells [28]. This is consistent with the abundance of H3K4me1 in the gene-rich EC1 and EC2 chromatin states and also with its presence in the gene-poor EC4 state contrasting its absence in the heterochromatin state C4 (Fig. 2). There is another histone modification, namely H3K27me3, that distributes quite differently in pluripotent and differentiated cells. In ESCs, this surrogate of polycomb activity is narrowly distributed (37% coverage) relative to the much broader distributions of H3K27me3 in K562 (54%), Monocd14ro1746 (65%), Gm12878 (55%), Hmec (54%) and Nhdfad (60%). In pluripotent cells, H3K27me3 is known to be mainly confined to “bivalent” promoters that also carry H3K4me3 [32–36, 112]. As indicated by the co-presence of H3K27me3 and H2AZ in the bivalent chromatin state EC2 (Fig. 2) and the observed local surrounding of H3K27me3 marks by H2AZ variants in the H1hesc epigenetic landscape (Fig. 5), the highly dynamic pluripotent chromatin is likely refractory to polycomb facultative heterochromatin formation and spreading [9, 28]. The smaller mean size of EC4 blocks ($\bar{L}=301$ kb) in H1hesc as compared to C4 blocks in K562 ($\bar{L}=882$ kb), Hmec ($\bar{L}=610$ kb) and Nhdfad ($\bar{L}=894$ kb) (Table 3), suggests that the gene-poor H2AZ marked accessible EC4 chromatin is incompatible with the stable interactions involved in the H3K9me3 enriched HP1 heterochromatin compaction and spreading (Fig. 2). All the other histone marks known to be involved in transcription positive regulation, including H3K27ac and H3K36me3, have a similar distribution with a similar coverage of the gene-rich genome regions in H1hesc (EC1+EC2) and differentiated cells (C1+C2) (S1 Table).

### Distinct epigenetic mechanisms of heterochromatin expansion during differentiation

There are mainly two epigenetic mechanisms of heterochromatin expansion during differentiation that correspond to the transitions towards the polycomb repressed state C2 and towards the HP1-associated heterochromatin state C4 (Fig. 3A). For the former mechanism, there are indeed two possible scenarios according to whether the pluripotent chromatin state that switches to C2 is EC2 or EC4. As previously described, EC2 is a bivalent chromatin state that is enriched in gene promoters that carry both the active mark H3K4me3 and the polycomb associated mark H3K27me3. This second mark has repressive effect on gene expression and contributes to maintain repression of bivalent genes including developmental genes [32–36, 112]. Some of these bivalent genes get activated during differentiation and switch from EC2 to the open euchromatin state C1 (Fig. 3A). The other ones experience some repression to the facultative chromatin state C2 via the expansion of H3K27me3 to often cover the entire gene and frequently neighboring gene loci [9]. But there is another category of genes that face this facultative heterochromatization which are the genes that are in the H2AZ rich accessible chromatin state EC4. These EC4 regions are actually lying nearby EC2 regions and get involved in the repressive expansion of H3K27me3 which dictates their switch to the polycomb repressed state C2. Note that this H3K27me3 spreading over several kb or tens of kb is locally at the expense of H2AZ which confirms that, in pluripotent cells, this histone variant is refractory to the compaction associated with polycomb repression [28]. Interestingly, the polycomb repressed scenario from EC4 to C2 mainly corresponds to MRT changes from late to early replicating loci (data not shown).

The second mechanism corresponds to transitions from the silent unmarked (EC3) and H2AZ rich accessible (EC4) states to the HP1-associated heterochromatin state C4 (Fig. 3A). This mechanism corresponds to a dramatic redistribution of the histone modification H3K9me3 which, although present in the pluripotent EC4 state, expands into large (from several 100 kb to a few Mb) late replicating highly compacted heterochromatin (Table 3, Figs. 4 and 5). H3K9me3 is important for the formation of the constitutive heterochromatin via the anchoring of the *α* and *β* isoforms of the HP1 protein [142, 143]. There is also evidence of some crosstalk between H3K9 methyltransferase (HKMT) and DNA methyltransferase (DNMT) [144, 145] that might explain the correlation observed between H3K9me3 and DNA methylation and the contribution of the later to the long-term maintenance of these large domains of late replicating C4 heterochromatin devoid of H2AZ and of any other histone modification but H3K9me3 [4, 9]. Knockout studies of H3K9 methyltransferases and H3K27 methyltransferases have led to differentiation or development defects [146–151], confirming that the epigenetic mechanisms underlying heterochromatin expansion play a critical role in cell fate determination.

### Master replication origins at U/N-domain borders are determinants of cell-fate commitment

**Figure 9:**
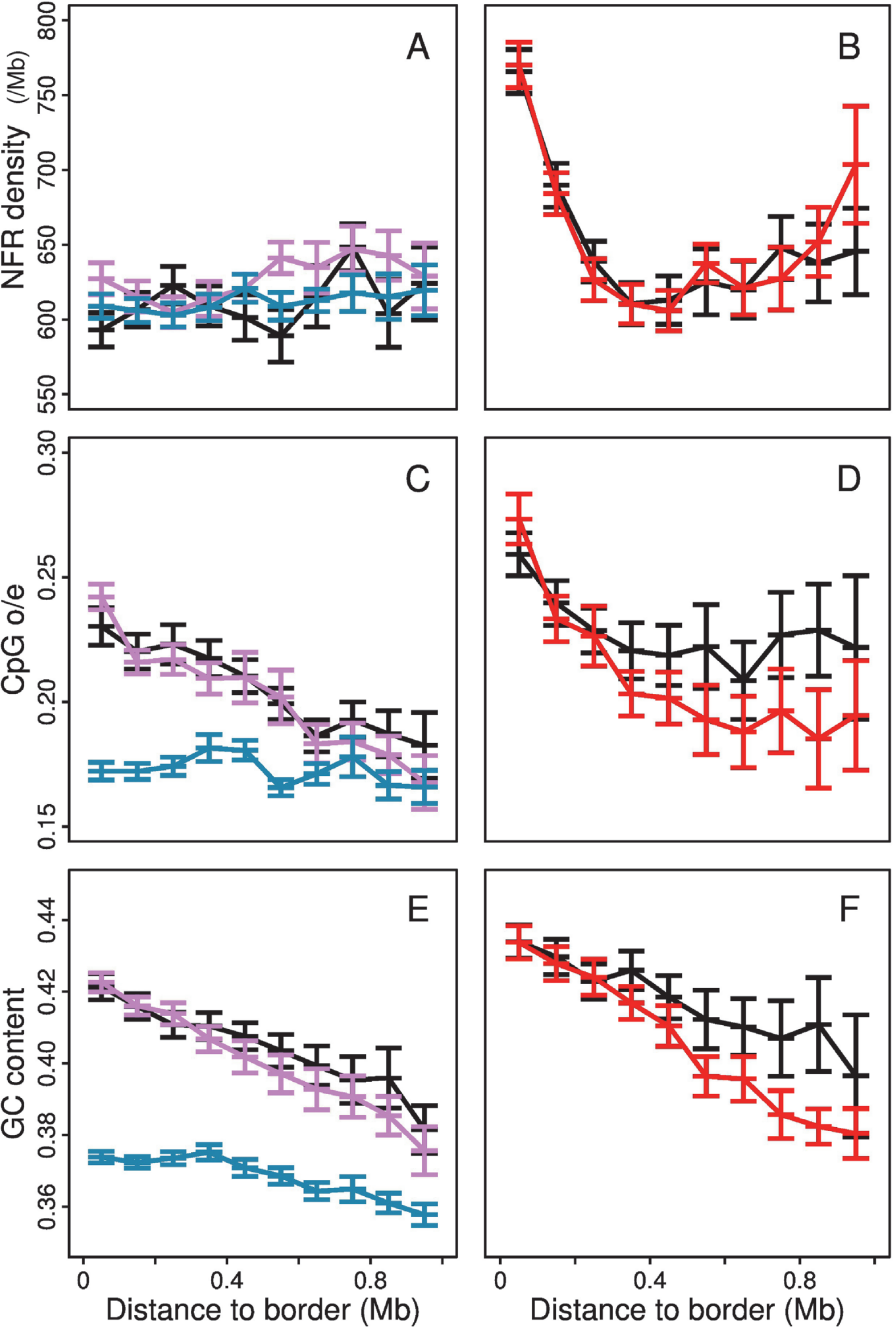
Sequence characterictics of MRT U-domains of H1hesc and Nhdfad. (A) Density of nucleosome free regions (NFRs) with respect to the distance to the closest U-domains border specific (n = 1) to the cell line. The different colors correspond to specific U-domains of Nhdfad (black), specific U-domains of H1hesc whose border is in EC1 or EC2 (red) and specific U-domains of H1hesc whose border is in EC4 (blue). (B) Density of (NFRs) with respect to the distance to the closest conserved (n = 6) U-domains border. (C) same as (A) for the CpG o/e. (D) same as (B) for the CpG o/e. (E) same as (A) for the GC content. (F) same as (B) for the GC content.

We found that MRT changes induced by differentiation resulted in an important change in the number and size of replication U/N-domains [77]. Small neighboring U/N-domains merged to become one large coordinately replicated domains (2 and 3 domains merged to 1 in Fig. 5 left and right column respectively). This replication domain consolidation [57, 58] is thus the consequence of an active early replication initiation zone in ESCs that no longer fires early in somatic cells. To characterize this consolidation phenomenon from pluripotent to differentiated cell lines as well as between differentiated cell lines, we defined an index of conservation *n* (Materials and Methods) that quantifies the number of U-domain borders in a given cell line that were also shared by *n*—1 other cell lines. To the sets of U-domains of the cell types considered so far, we added those previously identified in HeLa cells [61, 77] and the germline replication skew N-domains [77–83] (Fig. 5). For each cell type, about half U-domains are shared by at least another cell line, namely H1hesc (38.4%), K562 (61%), Gm12878 (59.2%), Nhdfad (51.6%) and Ndom (50.2%). Note that the smallest matching percentage was obtained for H1hesc as a direct consequence of the largest number of U-domains in this ESC line. When looking at U-domain borders individually (peaks in replication timing [61]), we got the following percentages of matching with at least another U/N-domain borders in another cell line: H1hesc (78.8%), K562 (88.1%), Gm12878 (88.9%), Nhdfad (85.6%) and Ndom (87.9%). As originally revealed in skew N-domains [51, 88] and further confirmed in MRT U-domains [77], there exists a remarkable gene organization inside these replication domains that turns out to be robust in each cell type. Expressed genes are confined in the euchromatin C1 (resp. EC1) environment of the bordering master replication origins whereas non expressed genes are distributed rather uniformly inside these domains (S8 Fig.) independently of the gradient of chromatin states (Fig. 7A’) (resp. Fig. 7A). When comparing the gene content nearby replication U/N-borders for increasing index of conservation (S8C Fig.), we found that the density as well as the distribution of non-expressed genes were quite insensitive to the degree of ubiquitiness of the nearby master replication origin. In other words, non-expressed genes seem to have no knowledge of the replication wave initiating at U/N-domain borders. We got the opposite for expressed genes with a significant enhancement of gene density when increasing the conservation index *n* (S8C Fig.). Ubiquitous master replication origins are surrounded by a C1 euchromatin environment which is hypomethylated (Fig. 9D), GC-high (Fig. 9F, Table 4), significantly enriched in DHS and CTCF (Table 4) and more importantly in nucleosome free regions (NFRs) (Fig. 9B) coded in the DNA sequence via high energy barriers that impair nucleosome formation (Material and Methods) [87, 104–108]. Thus these ubiquitous master replication origins are specified by an open chromatin structure which is to some extend encoded in the DNA sequence [3, 87]. This also provides some understanding of the local clustering of highly expressed genes with strong CpG rich promoters including house-keeping genes (S8B Fig.). As exemplified with the Nhdfad cell line, master replication origins that are specific to a differentiated cell line are still GC high (Fig. 9E) but no longer enriched in NFRs (Fig. 9A) suggesting that these early firing regions are epigenetically regulated and no longer favored by the DNA sequences. Indeed, Nhdfad specific master replication origins are hypomethylated (Fig. 9C), and significantly enriched in H2AZ (Fig. 8A) and CTCF (Fig. 8B) epigenetic marks. They are mainly surrounded by tissue specific genes with weak CpG poor promoters. Our results are consistent with previous reports that most genes do not change expression during domain consolidation in the MRT profile [57, 58, 60, 65]. When examining the joint distribution of gene expression in H1hesc and K562 (S5C Fig.), for 100kb loci that experience a EtoL transition and reversely for those that change from LtoE, we confirmed that most (∼ 55%) genes lying in dynamic MRT regions do not change expression, suggesting that, at the 100 kb scale, phenotypic differences between cell types are better reflected by epigenetic properties including the MRT than by transcriptional differences.

**Table 4:** Sequence, epigenetic and gene characteristics of conserved (n = 6) replication U-domain borders of H1hesc that switch from state ECi to Cj.

	Nb	GC	CpGo/e	H2AZ	CTCF	NANOG	OCT4	DHS	Promoters/border	Expressed promoters/border
1 to 1	38	0.445	0.233	0.331	0.663	1.321	0.335	0.016	3.947	3.237
1 to 2	3	0.458	0.235	0.346	0.472	0.733	0.145	0.018	3.333	3
2 to 1	4	0.444	0.198	1.214	1.066	1.254	0.133	0.025	3	1.75
2 to 2	6	0.427	0.206	0.606	0.872	0.556	0.458	0.021	2.667	2
2 to 3	1	0.436	0.179	0.468	0.248	0.277	0	0.014	2	2
3 to 2	1	0.418	0.16	0.765	0.419	0	0	0.008	3	3
4 to 2	1	0.391	0.143	0.526	0.261	0	0	0	2	2
4 to 3	1	0.445	0.192	1.227	0.049	0.234	0	0.004	1	1

For each border type, the table provides number (Nb), G+C content (GC), coverage by significantly enriched intervals relative to the genome average coverage for H2AZ (genome average 33%), CTCF (4.5%), NANOG (0.2%) and OCT4 (0.07%), coverage by DNase I hypersensitive sites (DHS), as well as the numbers of promoters and expressed promoters per border.

### ESC specific master replication origins as the cornerstone of pluripotency maintenance

Master replication origins that are specific to the pluripotent H1hesc cell line actually correspond to lineage-independent switches in MRT that are stably maintained after the late epiblast stage. These pluripotent master replication origins (N = 483) are almost equally distributed in the chromatin states EC1 (N = 113), EC2 (N = 131) and EC4 (N = 149) and only few are in the unmarked state EC3 (N = 51) and in the discarded set D (N = 41) (Table 5). Those that are gene rich in EC1 and EC2 environments display very similar properties than master replication origins specific to differentiated cell lines. They are hypomethylated (Fig. 9C), enriched in CTCF (Fig. 8B) and DHS (Table 5), their GC content is high (Fig. 9E) but they are not enriched in constitutive NFRs (Fig. 9A) as an indication of epigenetic regulation. Interestingly, these H1hesc specific (EC1, EC2) master replication origins are enriched in the key pluripotency transcription factors NANOG (Fig. 8C) and OCT4 (Fig. 8D) (Table 5). Note that they are also highly covered by the H2AZ mark but they are nonetheless depleted compared to the very high level coverage of the genome (Fig. 8A). Somatic specific master replication origins have the same coverage in H2AZ than specific ESC ones, but in contrast they are enriched compared to the genome background (Fig. 8A).

**Table 5:** Sequence, epigenetic and gene characteristics of specific (n = 1) replication U-domain borders of H1hesc that switch from state ECi to Cj.

	Nb	GC	CpGo/e	H2AZ	CTCF	NANOG	OCT4	DHS	Promoters/border	Expressed promoters/borde
1 to 1	58	0.442	0.226	0.361	0.602	1.129	0.159	0.014	3.379	2.655
1 to 2	15	0.429	0.21	0.44	0.655	2.689	0.759	0.013	1.667	1.4
1 to 3	12	0.42	0.201	0.241	0.488	0.998	0.199	0.013	1.917	1.25
1 to 4	14	0.399	0.194	0.469	0.212	0.783	0.13	0.009	0.643	0.643
2 to 1	9	0.418	0.224	0.672	0.886	1.474	0.076	0.019	1.889	1
2 to 2	56	0.441	0.205	0.804	0.903	1.507	0.355	0.017	2.357	1.25
2 to 3	16	0.43	0.181	0.582	0.482	2.418	0.691	0.014	0.875	0.5
2 to 4	19	0.408	0.214	0.977	0.503	2.968	0.76	0.012	0.684	0.158
3 to 2	5	0.374	0.15	0.276	0.336	0.074	0	0.003	1.4	0.6
3 to 3	11	0.371	0.143	0.228	0.069	0.257	0	0.003	0.273	0.273
3 to 4	14	0.352	0.165	0.352	0.068	0.217	0.165	0.004	0.214	0.143
4 to 1	1	0.399	0.231	0.189	0.288	0	0.389	0.008	0	0
4 to 2	13	0.391	0.155	0.824	0.314	0.244	0.148	0.008	1	0.308
4 to 3	22	0.388	0.164	1.007	0.226	0.577	0.257	0.006	0.864	0.409
4 to 4	46	0.376	0.172	0.984	0.14	1.232	0.408	0.006	0.174	0.087
4 to 4 intergenic	39	0.374	0.17	1.013	0.145	1.402	0.481	0.006	0	0

The information provided for each border category is the same as in Table 4.

More surprising is the non negligible proportion (30.9%) of specific H1hesc origins that belong to a EC4 environment and that mainly consolidate into a C4 heterochromatin domain (Table 5). These EC4 master replication origins indeed correspond to the early replicating EC4 regions that experience a EtoL transition mostly towards the HP1-associated heterochromatin state C4 (Table 5). They have totally different epigenetic and sequence properties. They are methylated (Fig. 9C), no longer enriched in CTCF (Fig. 8B) and DHS (Table 5), their GC content is low (Fig. 9E) and they are still not enriched in constitutive NFRs (Fig. 9A). Actually they are mainly epigenetically regulated by a local enrichment of H2AZ (Fig. 8A) that turns out to play an unexpected specific role in regulating the spatio-temporal replication program in pluripotent cells. Notably, these pluripotent specific EC4 master replication initiation zones are gene deserts: only 20/82 (∼ 24%) contain a gene promoter as compared to 82/99 (resp. 75/100) for those in EC1 (resp. EC2). Nevertheless, they are enriched in NANOG and OCT4 (Fig. 8C and D, Table 5), even in the intergenic ones, which suggests that these transcription factors are also involved in the regulation of replication in pluripotent cells. Note that the unusual principle of chromatin folding during development reported in [70] likely results from the C4 domain consolidation of these early ESC specific EC4 master replication origins (see for example one of them at position 47 Mb on the right panels of Fig. 5). As discussed in previous works [57, 58, 68, 70], the EtoL transitions associated with the consolidation of pluripotent specific EC1 (see for example one of them at position 12.5 Mb on the left panel of Fig. 5), EC2 and EC4 to HP1-associated C4 heterochromatin likely coincide with the emergence of compact chromatin near the nuclear periphery and with a dramatic large-scale 3D genome reorganization that may constitute an epigenetic barrier to cellular reprogramming. In that respect, the master-replication origins bordering ESC specific replication U/N-domains are likely to be major determinants in the maintenance of pluripotency.

### Conclusion/Perspectives

In summary, the integrative analysis of genome-wide epigenetic marks, expression and MRT data at 100 kb resolution in an ESC and several differentiated human cell lines, shows that the combinatorial complexity of these epigenetic data can be significantly reduced consistently with previous studies in *Drosophila* [66, 97], *Arabidopsis* [95] and *human* [28, 92, 99]. The epigenetic landscapes of pluripotent and differentiated cells are different even though, in both cases, four but distinct prevalent chromatin states are enough to characterize the diversity in chromatin environment along human chromosomes. Among these four states, only one is transcriptionally active and three are silent. The first one is a gene rich euchromatin state that is shared by pluripotent (EC1) and differentiated (C1) cells as well as the “unmarked” states EC3 and C3 that correspond to a silent state not enriched in any available epigenetic marks. The two other states are different as the signature of the global accessible character of the pluripotent chromatin [26]: H2AZ and H3K4me1 marks are broadly distributed [28] in the bivalent state EC2 containing bivalent genes and in the gene-poor accessible EC4 state as compared to the polycomb repressed state C2 and the HP1-associated heterochromatin state C4 that respectively result from the spreading of H3K27me3 and H3K9me3 in differentiated cells [9, 28]. When looking at the way these chromatin states are distributed along human chromosomes with a special focus on the regions where the MRT changes significantly during differentiation, we show that the master replication origins that border megabase-sized MRT U/N-domains [77, 78] are major determinants in cell-fate commitment and lineage fidelity. The minority (5.3%) that are conserved in all cell lines have a peculiar high GC hypomethylated (EC1, C1) euchromatin environment highly enriched in open marks including H2AZ, CTCF, DNase HS and also in NFRs encoded in the DNA sequence suggesting that these ubiquitous master replication origins have been selected during evolution. In these particularly highly decondensed regions are also found numerous CpG rich promoters of highly expressed genes including house-keeping genes. Most of the master replication origins that are cell type specific or shared by a few cell types, still correspond to GC-rich euchromatin mainly regulated epigenetically and no longer favored by a local abundance of NFRs encoded in the DNA sequence. They are mainly surrounded by highly expressed tissue-specific genes. A majority of master replication origins specific to ESCs have rather similar epigenetic properties with a high density of neighboring genes that are likely regulated by the pluripotency factors NANOG/OCT4. But what our study has revealed is the existence of a class of ESC specific master replication origins that fire early in a GC-low, gene desert EC4 environment and that experiences a change to a compact HP1-associated C4 heterochromatin environment during differentiation. These master origins have a specific epigenetic regulation that sheds a new light on the unexpected role of both H2AZ and the transcription factors NANOG/SOX2/OCT4 in the maintenance of the replication spatio-temporal program in pluripotent cells. An important proportion (67.4%) of the ESC specific master replication origins indeed correspond to EtoL transitions likely associated with some repositioning towards the nuclear periphery and some large-scale 3D chromatin rearrangements that may hinder cell reprogramming [57, 58, 68, 70]. As reported in previous studies of 4C [75] and Hi-C [77, 90] data in differentiated cell lines, these master replication zones at MRT U/N-domain borders act on the one hand as insulators that delimit topological domains of self-interacting chromatin [152], and on the other hand as long-distance interconnected hubs in the intra- and inter- chromosome interaction network [119]. This highlights the interest of performing a similar comparative analysis of Hi-C data in ESCs. Besides confirming the key role played by ESC specific master replication origins in the 3D chromatin regulation and control of pluripotency, it will likely bring new elements of discussion concerning the hypothetized influence of longer G1-phase enabling targeting of loci to the nuclear periphery and providing more time for nuclei to reorganize their genome before replication initiates in differentiated cells [57, 58]. As fundamental structural and functional units underlying the plasticity of replication domain organization in relation to gene expression and chromatin states, the replication timing U/N-domains together with the bordering master replication origins provide a framework for further studies in different cell types and different organisms, in both health and disease.

## Supporting Information

S1 Table Genome coverage by epigenetic marks in ESCs and differentiated cells. Percentage of 100-kb windows that contain a given epigenetic mark. (TIFF)

S1 Fig Spearman correlation matrix between epigenetic marks in Hmec, Monocd14ro1746, K562 and Gm12878 cell lines. Same color map as in Fig. 1.
(EPS)

S2 Fig PCA analysis and clustering procedure for ESC line (A-C) and the five differentiated cell lines (A’-C’). (A, A’) Percentage of variance accounted by the eleven principal components ordered according to their corresponding variance (eigenvalues). (B, B’) Scatterplot of the data points when projected on the (PC1, PC2) plane; color dots indicate the four chromatin states as found by our clustering procedure. (C, C’) Density of data points on the (PC1, PC2) plane using the same color coding as in (B, B’). In (B, C) the colors have the following meaning: EC1 (light pink) transcriptionally active chromatin, EC2 (light orange) bivalent chromatin, EC3 (light green) silent unmarked chromatin, EC4 (light blue) dynamically accessible chromatin poised to HP1-heterochromatin expansion. In (B’, C’) the colors correspond to: C1 (pink) transcriptionally active chromatin, C2 (orange) chromatin repressed by polycomb, C3 (green) silent unmarked chromatin, C4 (blue) HP1-associated heterochromatin. In (B, B’, C, C’) the points in dark grey are not classified in any chromatin state (Materials and Methods).
(EPS)

S3 Fig Repartition of the histone modifications H3K4me2, H3K79me2 and H4K20me1 in the four prevalent chromatin states of H1hesc cell line (EC1, EC2, EC3, EC4, same color coding as in S2B and C Fig.) and differentiated cell lines (C1, C2, C3, C4, same color coding as in S2B’ and C’ Fig.). Boxplots of the decimal logarithm of epigenetic mark CHip-seq read density in 100 kb non-overlapping windows per chromatin state.
(EPS)

S4 Fig Mosaic plots representing the probabilities of transition between the chromatin states of two different cell lines (from line 1 to line 2). The width of columns corresponds to the proportion of chromatin states in line 1. The segmentation for the *i* ^*th*^ column follows the proportion of windows in state (E)Ci in line 1 that become Cj in line 2. In other words, if we take the first pink rectangle of the first column, its width is proportional to the probability for a 100 kb window to be in chromatin state (E)C1 in line 1 and its height is proportional to the the probability for a 100 kb window to be in C1 in line 2 given that it is in (E)C1 in line 1. The area of this rectangle (product of the previously mentioned probability) is proportional to the probability for a window to be in state (E)C1 in line 1 and C1 in line 2.
(EPS)

S5 Fig Gene expression in the H1hesc and K562 chromatin states. (A) Density of promoters in the 4 chromatin states of the H1hesc cell line as a function of gene expression (genes were grouped into bins of width 0.05 in log_10_(RPKM) unit). Same color coding as in S2B and C Fig. (B) Density of promoters in the 4 chromatin states of the K562 cell line as a function of gene expression. Same color coding as in S2B’ and C’ Fig. (C) 2D representation of the joint density of gene expression in H1hesc (X-axis) and K562 (Y-axis) when focusing on EtoL (blue) and LtoE (magenta) MRT transitions. For comparison is shown as a control (black), the joint density obtained for comparable size sets of randomly chosen genes.
(EPS)

S6 Fig Spatial organization of chromatin states in H1hesc. (A) Histogram of chromatin state (EC1, EC2, EC3, EC4) block length in a logarithmic representation (Materials and Methods). (B) same as (A) for chromatin blocks formed by states EC1 and EC2 (EC1+EC2, light red) or by states EC3 and EC4 (EC3+EC4, light blue). (C) MRT in chromatin state blocks EC1+EC2 with respect to their length. Each 100 kb window in a chromatin state block is represented by the color of its state defined in S2B and C Fig. The mean profile was obtained by (i) ordering data points according to their block length, (ii) grouping them in classes of equal number of data points and (iii) computing the average length and MRT over each class. Vertical bars represent the standard deviation. Horizontal bars represent the range of length over each class. (D) Same as (C) for chromatin state blocks EC3+EC4.
(EPS)

S7 Fig Spatial organization of chromatin states in Nhdfad. (A) Histogram of chromatin state (C1, C2, C3, C4) block length in a logarithmic representation (Materials and Methods). (B) same as (A) for chromatin states C1 and C2 (C1+C2, light red) or by states C3 and C4 (C3+C4, light blue). (C) MRT in chromatin state blocks C1+C2 with respect to their length. Each 100 kb window in a chromatin state block is represented by the color of its state defined in S2B’ and C’ Fig. The mean profile was obtained by (i) ordering data points according to their block length, (ii) grouping them in classes of equal number of data points and (iii) computing the average length and MRT over each class. Vertical bars represent the standard deviation. Horizontal bars represent the range of length over each class. (D) Same as (C) for chromatin state blocks C3+C4.
(EPS)

S8 Fig Distribution of expressed (orange) and not expressed (blue) gene promoters inside replication timing U-domains of H1hesc (solid line) and Nhdfad (dashed line). (A) Mean density of gene promoters with respect to the distance to the closest U-domain border specific to the cell line (n = 1). (B) Mean density of gene promoters with respect to the distance to the closest U-domain common to all cell lines (n = 6). (C) Mean density of gene promoters in the 100 kb windows containing a U-domain border versus its conservation index n (Materials and Methods).
(EPS)

S9 Fig Spearman correlation matrix between epigenetic marks in HeLa. Same color coding as in Fig. 1.
(EPS)
