# Supplementary figures and images for: Embryonic Stem Cell Specific “Master” Replication Origins at the Heart of the Loss of Pluripotency
Source: PLoS Comput Biol. 2015 Feb 6;11(2):e1003969. doi: 10.1371/journal.pcbi.1003969 (PMC4319821; doi:10.1371/journal.pcbi.1003969)

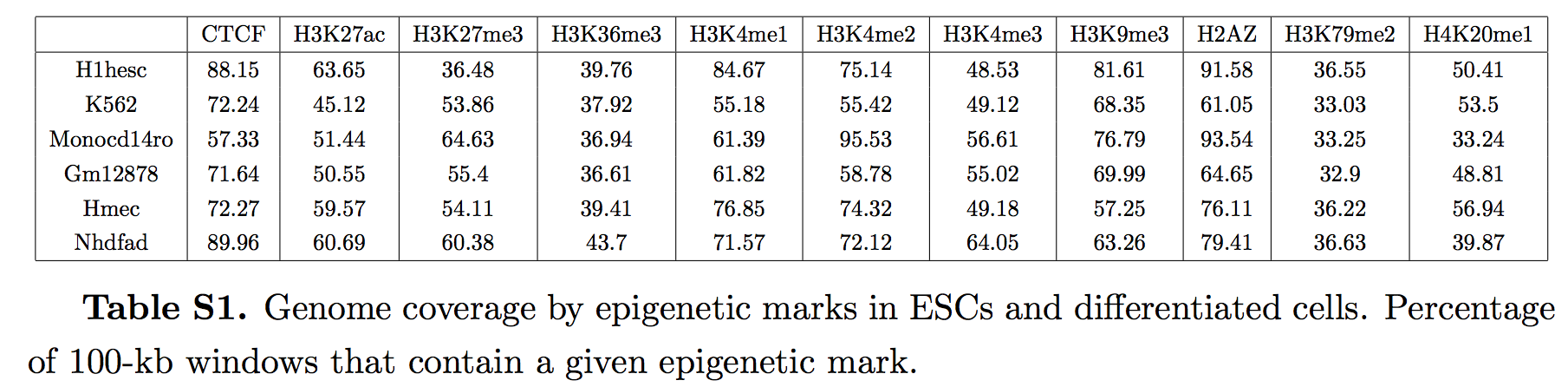

Supplement: S1 Table — (TIFF) [file pcbi.1003969.s001.tiff]
